# Formation of a low-symmetry Pd_8_ molecular barrel employing a hetero donor tetradentate ligand, and its use in the binding and extraction of C_70_†

**DOI:** 10.1039/d4sc01332h

**Published:** 2024-06-26

**Authors:** Dharmraj Prajapati, Jack K. Clegg, Partha Sarathi Mukherjee

**Affiliations:** a Department of Inorganic and Physical Chemistry, Indian Institute of Science Bangalore-560012 India psm@iisc.ac.in; b School of Chemistry and Molecular Biosciences, The University of Queensland St. Lucia Queensland 4072 Australia

## Abstract

The majority of reported metallo-supramolecules are highly symmetric homoleptic assemblies of M_*x*_L_*y*_ type, with a few reports on assemblies that are obtained using multicomponent self-assembly or using ambidentate ligands. Herein, we report the use of an unsymmetrical tetratopic ligand (L^un^) containing pyridyl and imidazole donor sites in combination with a *cis*-protected Pd(ii) acceptor for the formation of a low-symmetry M_8_L^un^_4_ molecular barrel (UNMB). Four potential orientational isomeric (HHHH, HHHT, HHTT, and HTHT) molecular barrels can be anticipated for the M_8_L^un^_4_ type metallo-assemblies. However, the formation of an orientational isomer (HHTT) of the barrel was suggested from single-crystal X-ray diffraction and ^1^H NMR analysis of UNMB. Two large open apertures at terminals and the hydrophobic confined space surrounded by four aromatic panels of L^un^ make UNMB a potential host for bigger guests. UNMB encapsulates fullerenes C_70_ and C_60_ favoured by non-covalent interactions between the fullerenes and aromatic panels of the ligand molecules. Experimental and theoretical studies revealed that UNMB has the ability to bind C_70_ more strongly than its lower analogue C_60_. The stronger affinity of UNMB towards C_70_ was exploited to separate C_70_ from an equimolar mixture of C_70_ and C_60_. Moreover, C_70_ can be extracted from the C_70_⊂UNMB complex by toluene, and therefore, UNMB can be reused as a recyclable separating agent for C_70_ extraction.

## Abstract 2

A tetradentate ligand with two different donor groups gave unsymmetrical molecular barrel UNMB*via* self-assembly. The stronger binding affinity of UNMB towards C_70_ over C_60_ was used to extract C_70_ from a mixture of C_60_/C_70_ in high purity.

## Graphical abstract

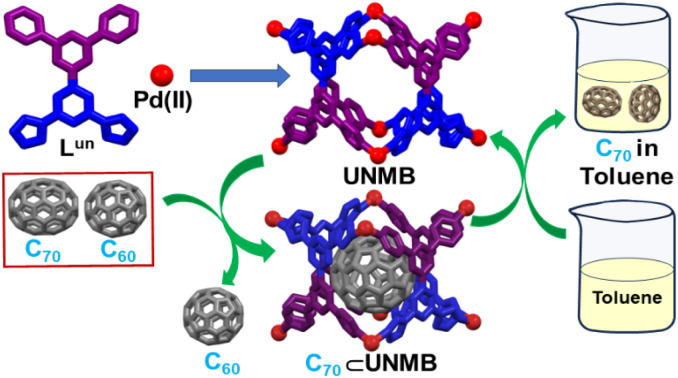

## Introduction

Nature has used various types of non-covalent interactions to control the preciseness of self-assembly processes to ensure that the individual components combine in specific ratios and orientations to function effectively.^1^ For instance, protein-based enzymes convene into homo- or hetero-polymeric quaternary structures to execute a variety of biological functions.^2^ The active sites of these enzymes are enclosed by low symmetrical and chiral cavities containing a combination of different chemical functionalities, such as catalytic sites, recognition sites, and conformational switches.^3^ Over the past few decades, chemists across the globe in different fields have tried to imitate these principles by applying them to synthetic systems.^4^ In this regard, a wide range of supramolecular architectures, such as self-assembled coordination cages,^5^ molecular capsules,^6^ and molecular barrels^7^ that demonstrate a vast range of important applications, have been developed.^8^ Nonetheless, the majority of such self-assembled molecules reported to date are highly symmetric and homoleptic complexes of M_*x*_L_*y*_ type, which were prepared by incorporating a selected metal node (M) and only one type of symmetric ligand (L).^5–8^ Recently, there has been a push towards unfolding lower symmetry systems intending to get upgraded functionally. In addition to heading towards supramolecular assemblies with reduced symmetry,^9^ such as heteroleptic cage systems^10^ and hetero-polymetallic cage systems,^11^ there has also been a rise in interest in developing self-assembled systems containing bis-monodentate ligands of two different donor groups.^12^ However, unsymmetrical multitopic ligands (number of donor sites > 2) with distinct binding sites remain largely ignored because of the possibility of the formation of a mixture of different isomers. An isomeric mixture of the complexes might be formed due to the random relative orientational arrangement of the ligands around the metal centres. Thus, selecting an unsymmetrical ligand with distinct donor sites for the synthesis of self-assembled coordination complexes of lower symmetry is quite challenging.

Fullerene is one of the stable allotropes of carbon and has a vast range of captivating properties, such as conducting, magnetic, antioxidant, and electronic, due to its unusual symmetry and extended conjugated electronic features.^13^ Owing to these properties, fullerenes find applications in several fields, for instance in materials science,^14^ in superconducting materials,^15^ as electroactive materials in solar cells,^16^ and for biological applications.^17^ However, these applications largely depend on their purity and solubility. Unrefined carbon soot contains a mixture of fullerenes of different carbon numbers and amorphous forms of carbon and other allotropes such as carbon nanotubes.^18^ The popular purification techniques to separate fullerenes from carbon soot are recrystallization, controlled sublimation, and extraction with organic solvents.^19^ In recent times, chromatographic methods have been used predominantly for the isolation and purification of fullerenes.^20^ Although efficient columns are available for the isolation of fullerene using HPLC techniques, all of these purification methods need large amounts of solvents and can induce irreversible adsorption, and decomposition of fullerene within the column.^20^ Moreover, these techniques are often expensive, tedious, energy and time-consuming, and in some instances, it is difficult to get a particular fullerene with high selectivity. Therefore, inventing techniques for the purification of fullerenes is a challenging and highly desirable task in materials chemistry. Over the past few decades, selective separation of fullerenes by encapsulation within soluble supramolecular receptors has attracted the research community's attention^21^ because this method offers potential selectivity *via* selective host–guest complexation without any special equipment. Moreover, the encapsulation of fullerene enhances its solubility. Therefore, it is highly appealing to devise a suitable molecular host that has a better binding affinity for one fullerene over the other, leading to their separation from a mixture.

**Scheme 1:**
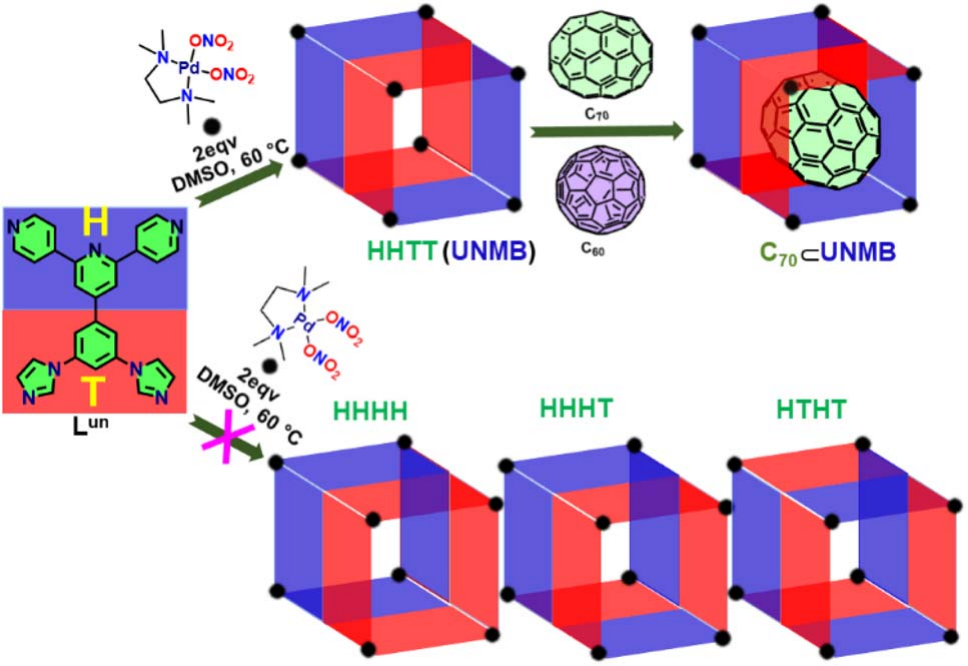
Schematic presentation of the synthesis of unsymmetrical molecular barrel UNMB, and its selective encapsulation of C_70_.

Herein, we report the formation of an unsymmetrical molecular barrel (UNMB) of M_8_L^un^_4_ type by coordination-driven self-assembly of an unsymmetrical tetratopic donor 4′-(3,5-di(1*H*-imidazole-1-yl)phenyl)-4,2′:6′,4′′-terpyridine (L^un^) containing pyridine and imidazole donor sites with *cis*-[(tmeda)Pd(ONO_2_)_2_] as an acceptor in DMSO (tmeda = *N*,*N*,*N*′,*N*′-tetramethyl-ethane-1,2-diamine) (Scheme 1). The orientational isomeric product (UNMB) was characterized by ^1^H NMR, 2D DOSY NMR, and ESI-MS analysis. Furthermore, the molecular structure was unambiguously established by single-crystal X-ray diffraction analysis. UNMB features a rhombohedral hydrophobic cavity fenced by extended π-conjugated aromatic rings of the four ligand units (L^un^) along with two large open windows. Above-mentioned features of UNMB assist the encapsulation of C_70_ and C_60_ inside its hydrophobic cavity. ESI-MS analysis of C_70_⊂UNMB and C_60_⊂UNMB revealed the formation of 1 : 1 host–guest complexes (Scheme 1). Furthermore, association constant values (*K*_a_), DFT studies, and competitive guest encapsulation studies suggested that UNMB has better binding affinity towards C_70_ over C_60_, which enables the recyclable separation of C_70_ from an equimolar mixture of C_60_ and C_70_.

## Results and discussion

### Synthesis and characterization of UNMB

**Fig. 1:**
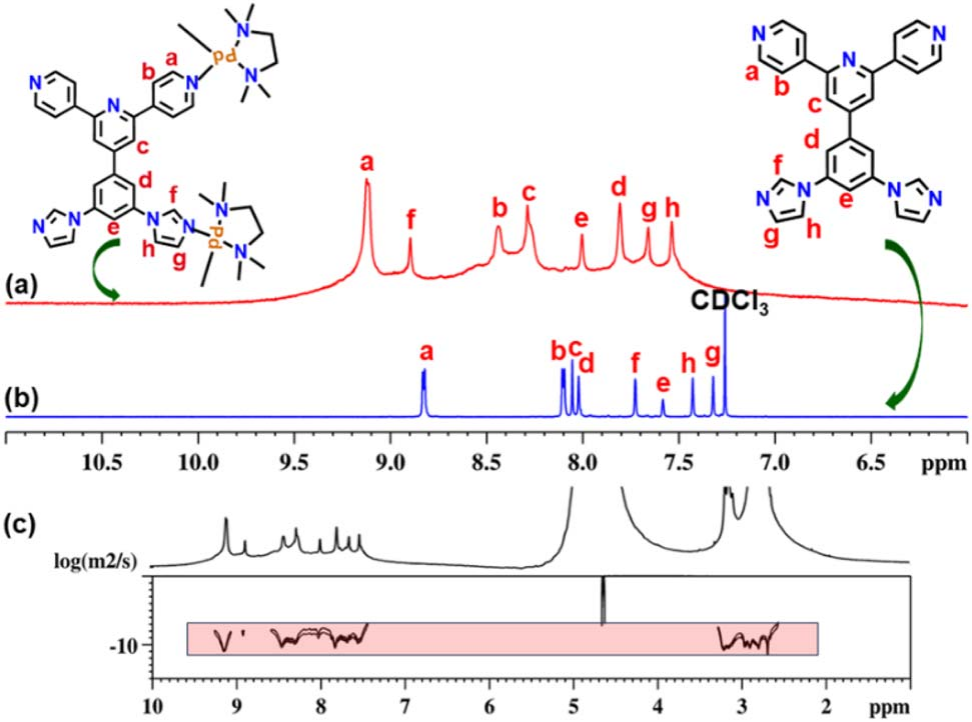
Stacked partial ^1^H NMR spectra of (a) UNMB in D_2_O, (b) ligand L^un^ in CDCl_3_, and (c) diffusion-ordered ^1^H NMR of UNMB in D_2_O.

The new tetratopic unsymmetrical ligand L^un^ containing two imidazole and two pyridyl donor sites was synthesized by standard Ullman coupling of 4′-(3,5-dibromophenyl)-4,2′:6′,4′′-terpyridine with imidazole (Scheme S1†).^20^ Ligand L^un^ was characterized by ^1^H and ^13^C NMR spectroscopy (Fig. S2–S5†). A mixture of ligand L^un^ (1 equivalent) and metal acceptor *cis*-[(tmeda)Pd(ONO_2_)_2_] (M) (2 equivalents) in DMSO was heated for 12 h at 60 °C under stirring. Afterward, the resulting clear solution was precipitated by treating it with an excess of ethyl acetate. The precipitate was centrifuged and dried under vacuum to get a white powder of UNMB in 98% yield. The obtained white powder of UNMB was analysed by NMR and ESI-MS spectroscopy. Due to the unsymmetrical nature of the tetratopic donor L^un^, upon self-assembly with 90° acceptor M, it may produce different orientational isomers of the most common compositions, such as M_6_L^un^_3_ or M_8_L^un^_4_. The ^1^H NMR spectrum of UNMB in D_2_O displayed a set of eight peaks in the aromatic region and three signals each for ethylene and methyl protons of the acceptor unit in the aliphatic region (2.6–3.3 ppm) (Fig. 1, S6 and S7†). The integral ratio (1 : 2 : 1) of the proton signals of ethylene and methyl groups in the aliphatic region probably hints at either the formation of the product that has three different kinds of (tmeda)Pd(ii) units in a 1 : 2 : 1 ratio (Fig. S8†) or an equilibrium mixture. Moreover, a significant downfield shift was observed in the signals of pyridyl α-protons (H_a_) and imidazole protons (H_f_) with Δ*δ* = 0.33 and Δ*δ* = 1.16 ppm, respectively, which indicates the formation of ligand-to-metal dative bonds (Fig. 1 and S6†). Additionally, the appearance of a single diffusion band in the 2D DOSY (diffusion order spectroscopy) NMR spectrum indicated the formation of a single self-assembled molecular architecture (Fig. 1c and S8†). Moreover, all the proton signals of UNMB were assigned by a thorough investigation of ^1^H–^1^H COSY NMR, which confirmed that all signals are originated from the ligand L^un^ (Fig. S9†). Thus, altogether, the NMR spectral data provided preliminary information in support of the formation of a self-assembled architecture, but due to the absence of the required splitting patterns of the proton signals, it could not suggest the arrangement of the donors in the final assembly.

**Fig. 2:**
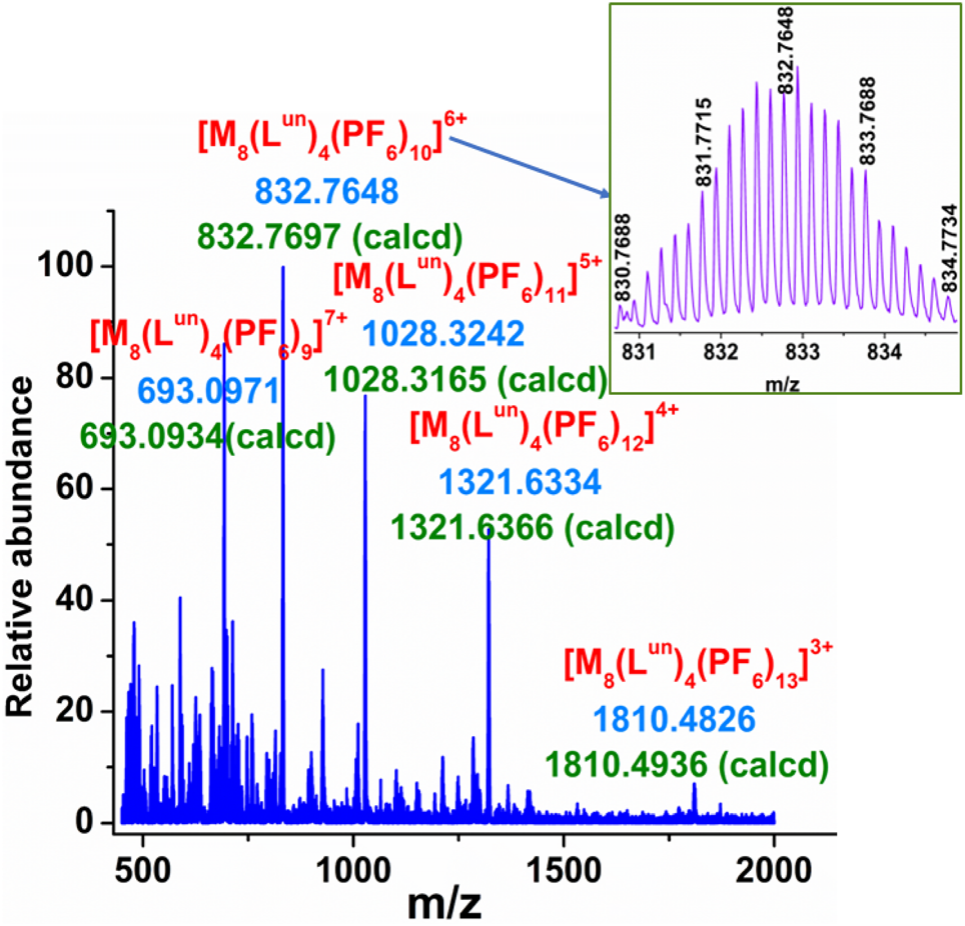
ESI-MS spectrum of the PF_6_^−^ analogue of UNMB in acetonitrile. (Inset) Experimental isotopic distribution pattern of the [M_8_L^un^_4_(PF_6_)_10_]^6+^ fragment.

The molecular composition of the self-assembled molecular architecture in solution was ascertained by electrospray ionization mass spectroscopy (ESI-MS). For this, an aqueous solution of UNMB was reacted with an excess of KPF_6_ at room temperature overnight. The ESI-MS spectrum of the PF_6_^−^ analogue was recorded in acetonitrile. The ESI-MS spectrum showed the presence of several noticeable peaks and respective isotopic distribution patterns corresponding to the charge fragments at *m*/*z* = 1810.4826 for [M_8_L^un^_4_(PF_6_)_13_]^3+^, 1321.6334 for [M_8_L^un^_4_(PF_6_)_12_]^4+^, 1028.3242 for [M_8_L^un^_4_(PF_6_)_11_]^5+^, 832.7648 for [M_8_L^un^_4_(PF_6_)_10_]^6+^, and 693.0971 for [M_8_L^un^_4_(PF_6_)_9_]^7+^, which are well matched with the respective calculated isotopic distribution patterns of the above mentioned charged fragments (Fig. 2, S11 and S12†). Thus, ESI-MS investigation suggested the formation of a molecular architecture with the composition of M_8_L^un^_4_.

Due to the different orientations of four molecules of non-symmetric ligand L^un^, several orientational isomeric barrels like HHHH, HHHT, HHTT and HTHT (Scheme 1) are possible. In the case of the most symmetric HHHH (having *C*_4V_ symmetry) isomer, there will be two kinds of palladium centres in a 1 : 1 ratio. One set of Pd ions will be coordinated to the pyridyl units and another set will be connected with the imidazole units; hence, in this case, the ^1^H NMR pattern will presumably be simpler.^7*e*,*f*^ However, in the case of the HTHT (having *D*_2d_ symmetry) isomer, there will be only one kind of Pd(ii) centre; hence, each pyridyl and imidazole unit will face an identical electronic environment, and therefore, again, a much simple NMR pattern is expected. The aliphatic proton peaks' integrations in a ratio of 1 : 2 : 1 hint at the formation of either the HHTT (having *C*_2v_ symmetry) or HHHT (having *C*_s_ symmetry) isomer. Only these two isomers have three different kinds of (tmeda)Pd(ii) units. ^1^H NMR data in combination with the ESI-MS result indicated the formation of one of these isomers.

**Fig. 3:**
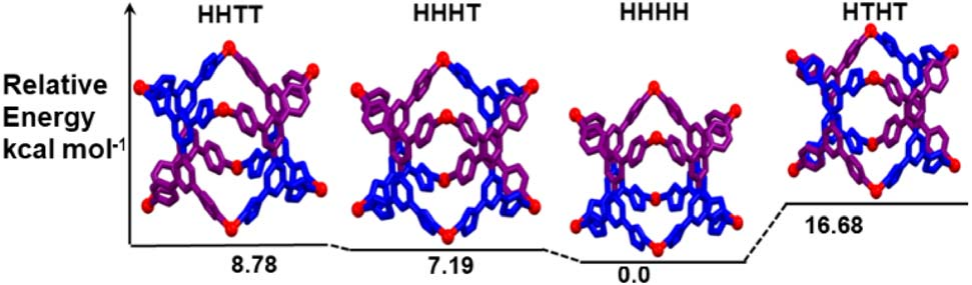
Energy level diagram with the DFT [B3LYP/LanL2DZ, 6-31G] geometry optimized structures in the gas phase for the four possible linkage isomers of the [Pd_8_(L^un^)_4_]^16+^ complex.

A computational study was performed to determine the comparative stabilities of these isomeric barrels, depicted in Scheme 1. Initially, the computational optimization for all the isomers was performed by the PM6 semiempirical method in their ground state. Next, single point energy calculations were carried out by employing the DFT method (B3LYP/LanL2DZ, 6-31G). From the DFT calculations, it can be observed that orientational isomer HHHH is found to be the energetically most favoured isomer, while isomer HTHT is the least favourable (Fig. 3). However, the energy difference among the HHHH, HHHT and HHTT isomers was found to be small (<20 kcal mol^−1^) (Table S2†). Moreover, the energy difference between the HHTT and HHHT isomers (one of which is indicated to be formed by ^1^H NMR analysis) is almost negligible. Thus, the theoretical study did not give enough information to predict the isomer formed.

**Fig. 4:**
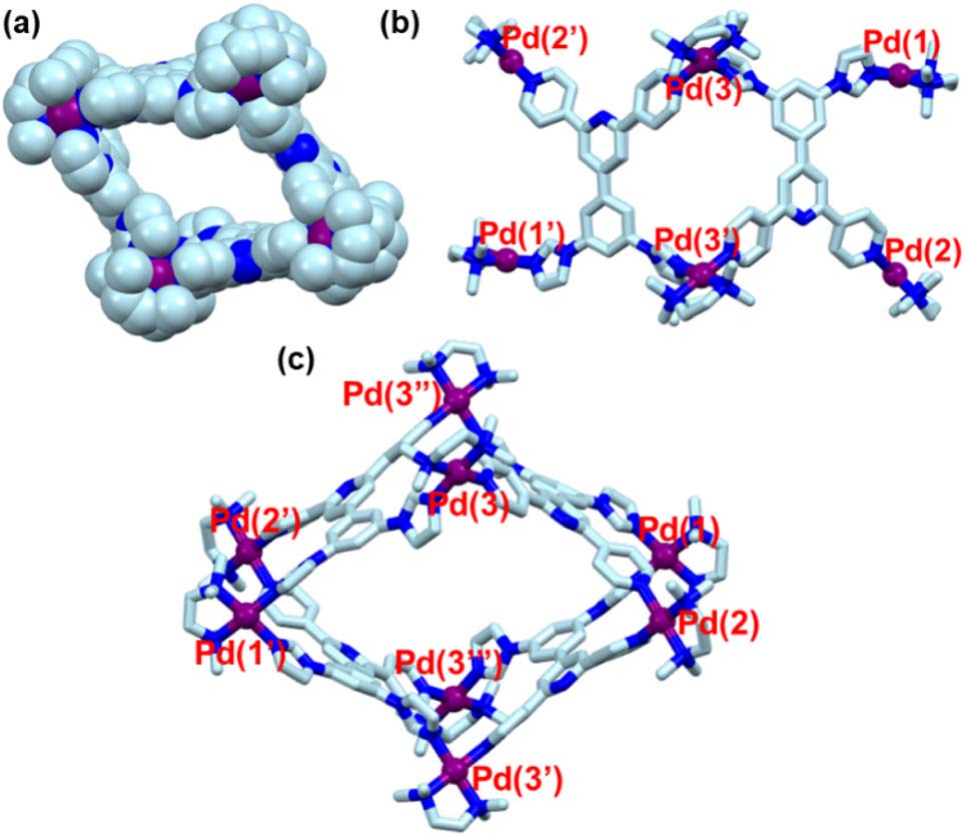
SC-XRD structure of UNMB: (a) space-filling diagram; (b) side view; (c) top view (capped stick model) [color codes: C (light blue), N (blue), Pd (purple)]. Counter anions, hydrogen atoms, and solvent molecules have been omitted for the sake of clarity.

Although ^1^H NMR and ESI-MS analyses gave a preliminary idea about the formation of a M_8_L^un^_4_ barrel, such studies could not predict the actual isomer formed. Therefore, to get precise information about the isomer formed, a single-crystal X-ray diffraction study was necessary. To do this, suitable single crystals were grown by slow diffusion of acetone vapour into an aqueous solution of UNMB at room temperature. The diffraction study was carried out with a synchrotron beam line.^22^ The single crystal data unequivocally revealed the formation of the HHTT isomer (Fig. 4).

The Pd_8_ barrel crystallized in the triclinic system with space group *P*1̄ and two molecules were found in the asymmetric unit. The crystal structure of UNMB revealed that there are three types of Pd(tmeda) units present in UNMB. Two Pd centres (labelled as Pd1 and Pd1′) out of eight relate to only imidazole rings and other set of Pd centres (labelled as Pd2 and Pd2′) are coordinated only with pyridine units, whereas remaining four Pd ions (labelled as Pd3, Pd3′, Pd3′′, and Pd3′′′) are each bound to one imidazole unit and one pyridine ring. This mode of coordination bonding between the Pd(tmeda) centres and imidazole/pyridine units of L^un^ resulted in orientational self-sorting type self-assembly and gave rise to the formation of the HHTT isomer. The presence of three different types of (tmeda)Pd(ii) units in the single crystal structure of UNMB thus supports the findings of the ^1^H NMR data. Moreover, the redissolved crystals gave a very clean ^1^H NMR spectrum, which exactly matches that of the as synthesized barrel UNMB. This finding further gives primary evidence for the formation of the HHTT isomer in solution. Therefore, single-crystal XRD analysis confirmed the formation of the low symmetry barrel HHTT. The average distance between the Pd(ii) ions in opposite corners is ∼21.25 Å for Pd1–Pd2′ and ∼15.65 Å for Pd3–Pd3′′; however, the distance between the adjoining Pd centres is roughly ∼12.67 Å.

### Fullerene encapsulation studies

**Fig. 5:**
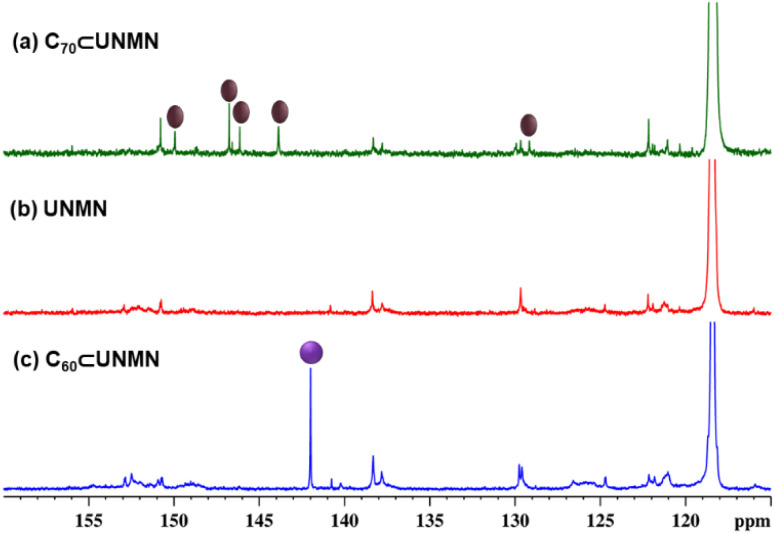
Stacked partial ^13^C NMR spectra of (a) C_70_⊂UNMB, (b) UNMB, and (c) C_60_⊂UNMB recorded in CD_3_CN at room temperature.

Two large open terminal windows and the hydrophobic cavity enclosed by the aromatic rings of four ligands make UNMB a suitable encapsulant for the entrapment of large guest molecules. Therefore, to investigate the guest binding ability of UNMB, we chose large sized insoluble guests C_60_ and C_70_. An aqueous solution (0.5 mL) of UNMB was stirred with 2 equivalents of fullerene C_60_ for 12 h at 55 °C, but the resultant solution showed no colour change. ^1^H, ^13^C and ESI-MS spectra showed the spectral data corresponding to only free UNMB. Evidently, there is no interaction between C_60_ and the host in an aqueous medium. Moreover, stirring C_70_ and UNMB did not result in any change in the spectral features, indicating no encapsulation of C_70_ either in an aqueous medium. Stunningly, when two equivalents of solid C_60_ were added to the acetonitrile solution of UNMB (PF_6_-analogue) and heated at 55 °C under stirring, the colour of the reaction mixture slowly changed to light violet within 2 h and it got darker after completion of the reaction. The resulting suspension was centrifuged, and the supernatant was examined by ESI-MS, ^1^H and ^13^C NMR and electronic absorption spectroscopy. As C_60_ is insoluble in acetonitrile, the appearance of the characteristic profile in the spectroscopic data suggested the binding of C_60_ with UNMB in an acetonitrile medium. In contrast to the sharp and simple signals in the ^1^H NMR spectrum of the free UNMB host, C_60_⊂UNMB displayed multiple signals suggesting strong enough interaction between the guest C_60_ and host UNMB (Fig. S13†). Additionally, a single horizontal band in the DOSY NMR spectrum revealed that all the signals belong to a single species (Fig. S14†). Moreover, a sharp and intense signal at 142 ppm was obtained in the ^13^C NMR spectrum of C_60_⊂UNMB due to C_60_, which further confirms the binding of C_60_ with UNMB (Fig. 5 and S15†).

**Fig. 6:**
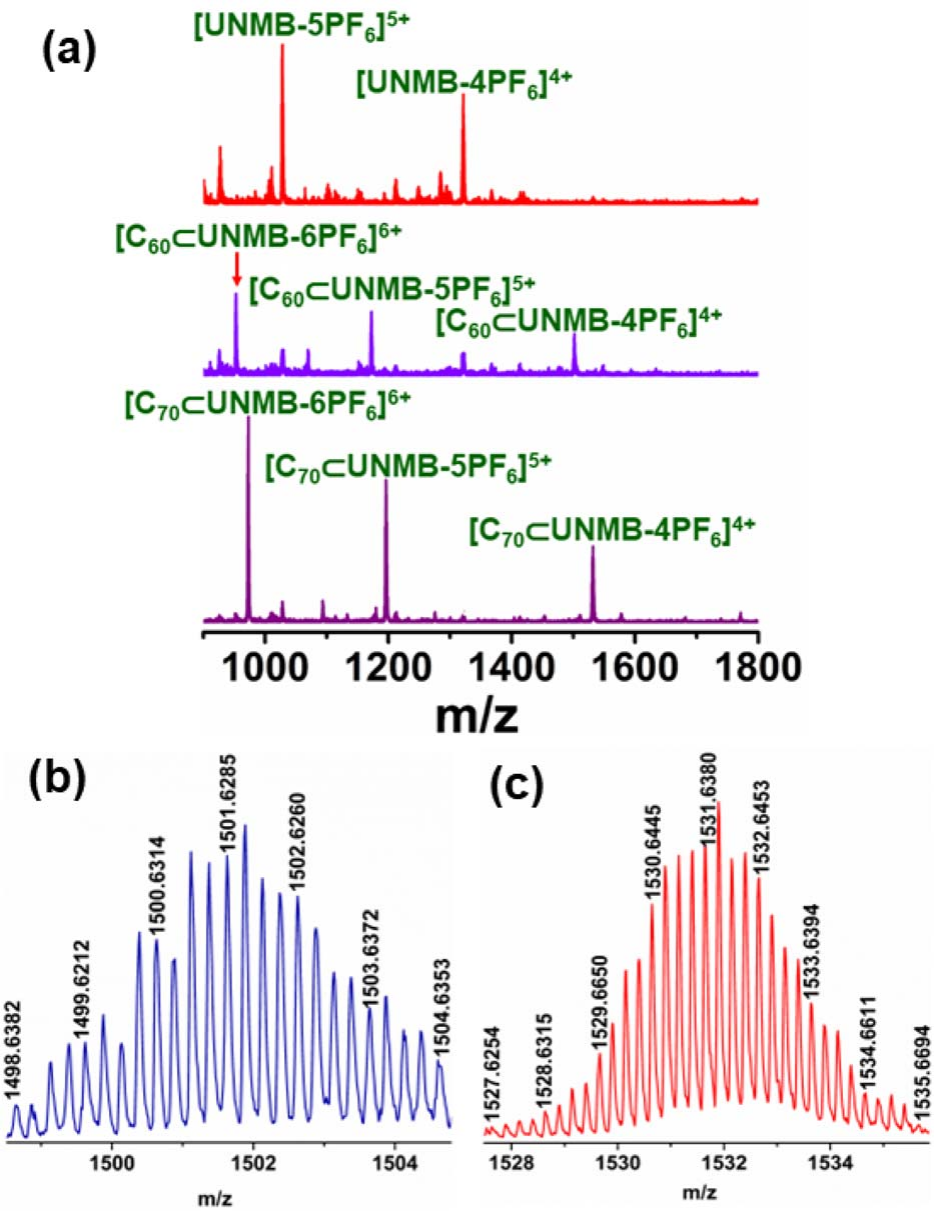
(a) Stacked partial ESI-MS spectra of UNMB (red), C_60_⊂UNMB (violet), and C_70_⊂UNMB (purple). Experimental isotopic distribution patterns of (b) [C_60_⊂UNMB(PF_6_)_12_]^4+^, and (c) [C_70_⊂UNMB(4PF_6_)_12_]^4+^ fragments in ESI-MS.

In line with this, the ESI-MS spectrum showed several peaks corresponding to charge fragments associated with host–guest complex C_60_⊂UNMB. The isotopic distribution pattern corresponded to charge fragments at *m*/*z* = 1501.6285 for [C_60_⊂UNMB(PF_6_)_12_]^4+^, 1172.3069 for [C_60_⊂UNMB(PF_6_)_11_]^5+^, 952.7668 for [C_60_⊂UNMB(PF_6_)_10_]^6+^, 795.9456 for [C_60_⊂UNMB(PF_6_)_9_]^7+^, and 678.3319 for [C_60_⊂UNMB (12PF_6_)_8_]^8+^ (Fig. 6, S16 and S17†), suggesting the formation of a host–guest adduct with the stoichiometry of 1 : 1. These isotopic distribution patterns matched well with the simulated isotopic distribution pattern for the respective charge fragments. Furthermore, the ESI-MS spectrum of the C_60_⊂UNMB adduct showed the presence of peaks for free UNMB in addition to the signals for C_60_⊂UNMB. This is owing to the decomplexation of the host–guest adduct at the time of ionization.

Similarly, host–guest complexation was performed employing C_70_ as a guest. A colourless acetonitrile solution of UNMB was treated with two equivalents of solid C_70_ at 55 °C under stirring, which resulted in a deep purple suspension after 12 h. After removal of the excess guest, the deep purple solution was analysed with various spectroscopic methods. Like C_60_⊂UNMB, C_70_⊂UNMB displayed multiple peaks in the ^1^H NMR spectrum (Fig. S18†); however, the single diffusion band in DOSY NMR spectroscopy revealed that all the signals are associated with a single species (Fig. S19†). Interestingly, five additional signals at 149.55, 146.59, 146.15, 143.87 and 129.16 ppm appeared in the ^13^C NMR of the C_70_⊂UNMB complex (Fig. 3 and S20†), contrary to the single extra peak in the case of the C_60_⊂UNMB adduct. The appearance of these new peaks is due to the presence of chemically different carbon atoms in C_70_,^22^ and the presence of these additional resonance peaks strongly suggests the formation of an inclusion complex of UNMB with C_70_.

The ESI-MS spectrum of C_70_⊂UNMB showed six isotopically well resolved peaks at *m*/*z* = 2090.5246, 1531.6380, 1196.3206, 972.7755, 813.0999 and 693.3494 corresponding to charge fragments [C_70_⊂UNMB(PF_6_)_13_]^3+^, [C_70_⊂UNMB(PF_6_)_12_]^4+^, [C_70_⊂UNMB(PF_6_)_11_]^5+^, [C_70_⊂UNMB(PF_6_)_10_]^6+^, [C_70_⊂UNMB(PF_6_)_9_]^7+^, [C_70_⊂UNMB(PF_6_)_8_]^8+^, respectively (Fig. S21 and S22†). The isotopic distribution pattern of these peaks resembled the theoretically simulated patterns, which supports C_70_⊂UNMB adduct formation in 1 : 1 host guest stoichiometry. In fact, in comparison to C_60_⊂UNMB, the ESI-MS data showed mainly peaks corresponding to C_70_⊂UNMB along with a trace quantity of the free host. This undoubtedly suggests the partial dissociation of C_70_⊂UNMB over the ionization period and advocates for the stronger binding ability of UNMB for C_70_ as compared to C_60_ (Fig. 6a).

**Fig. 7:**
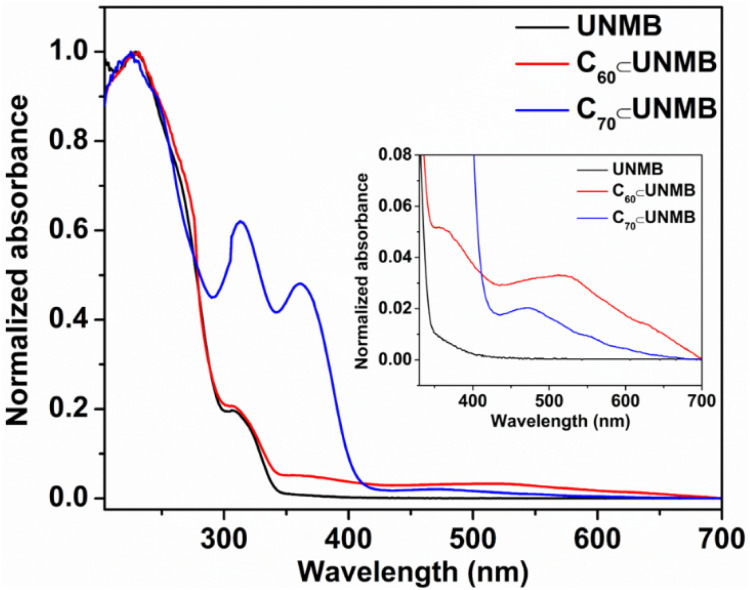
Absorption spectra of UNMB, C_60_⊂UNMB and C_70_⊂UNMB at room temperature in acetonitrile (10^−5^ M solution). Inset: enlarged UV-vis spectra of UNMB, C_60_⊂UNMB, and C_70_⊂UNMB showing the absorption band due to encapsulated fullerenes.

In line with this, we employed UV visible absorption spectroscopy to characterize the host–guest complexation. The UV-Vis absorption spectrum of ligand L^un^ in chloroform showed two absorption bands at *λ*_max_ 245 and 303 nm, which are due to π–π* transition (Fig. S23†). Meanwhile, the electronic absorption spectrum of UNMB displayed two absorption bands at *λ*_max_ 232 and 308 nm that can be ascribed to the π–π* transition originating from the L^un^ units (Fig. 7). The presence of additional broad bands at around *λ*_max_ = 362 and 520 nm is owing to C60⊂UNMB, and a strong absorption band at *λ*_max_ = 361 nm along with a broad band around *λ*_max_ = 473 nm is due to C_70_⊂UNMB in the respective UV-visible absorption spectrum. This truly speaks for the binding of fullerenes with the host UNMB (Fig. 7).^23^

After establishing the C_60_/C_70_-UNMB host–guest complexation qualitatively using NMR, ESI-MS and UV-vis analysis, the binding constants for their formation were determined by UV-vis titrations. Because of the insolubility of fullerenes in acetonitrile, stock solutions (1 mM) of the fullerenes were prepared in toluene. An acetonitrile solution (10 μM) of UNMB was titrated with the required amount of fullerene in toluene (Fig. S24 and S26†). Changes in absorbance at *λ*_max_ 306 nm were plotted against the number of equivalents of C_60_/C_70_ added, which suggested the formation of a 1 : 1 inclusion complex in both cases (Fig. S24 and S26†). The Benesi–Hildebrand plots (B–H plots)^24^ were used to calculate the binding constants, which were roughly found to be 7.15 × 10^5^ M^−1^ for C_70_⊂UNMB (Fig. S24 and S25†) and 2.83 × 10^4^ M^−1^ for C_60_⊂UNMB (Fig. S26 and S27†), which matches well with the literature reports.^18*c*^

Many attempts were made to grow suitable single crystals of fullerene⊂UNMB for SC-XRD diffraction, but they remained unsuccessful. Therefore, to obtain a clear idea about the host–guest interactions between the host UNMB and guest fullerenes, we did energy optimization of these host–guest complexes using the PM6 semiempirical method in the ground state. The optimized structure of the inclusion complexes revealed that fullerenes C_60_ and C_70_ fit perfectly within the pocket of host UNMB (Fig. S28†). The distance between the walls of the UNMB and C_60_ surface is ∼3.3 Å, whereas those of the C_70_ surface are ∼3.3 and 3.5 Å based on the two orientations of C_70_. The obtained values lie within the range of the required length for efficient π–π interaction between the host and guest.^25^ Furthermore, single point energy was computed by employing the DFT method for C_70_⊂UNMB and C_60_⊂UNMB. These theoretical studies exhibited that the host–guest complexation of C_70_⊂UNMB is energetically more stable than that of C_60_⊂UNMB. Thus, theoretical studies support the stronger affinity of UNMB towards C_70_ found by the association constant values.

### Selective extraction of C_70_ from a C_60_/C_70_ mixture

**Fig. 8:**
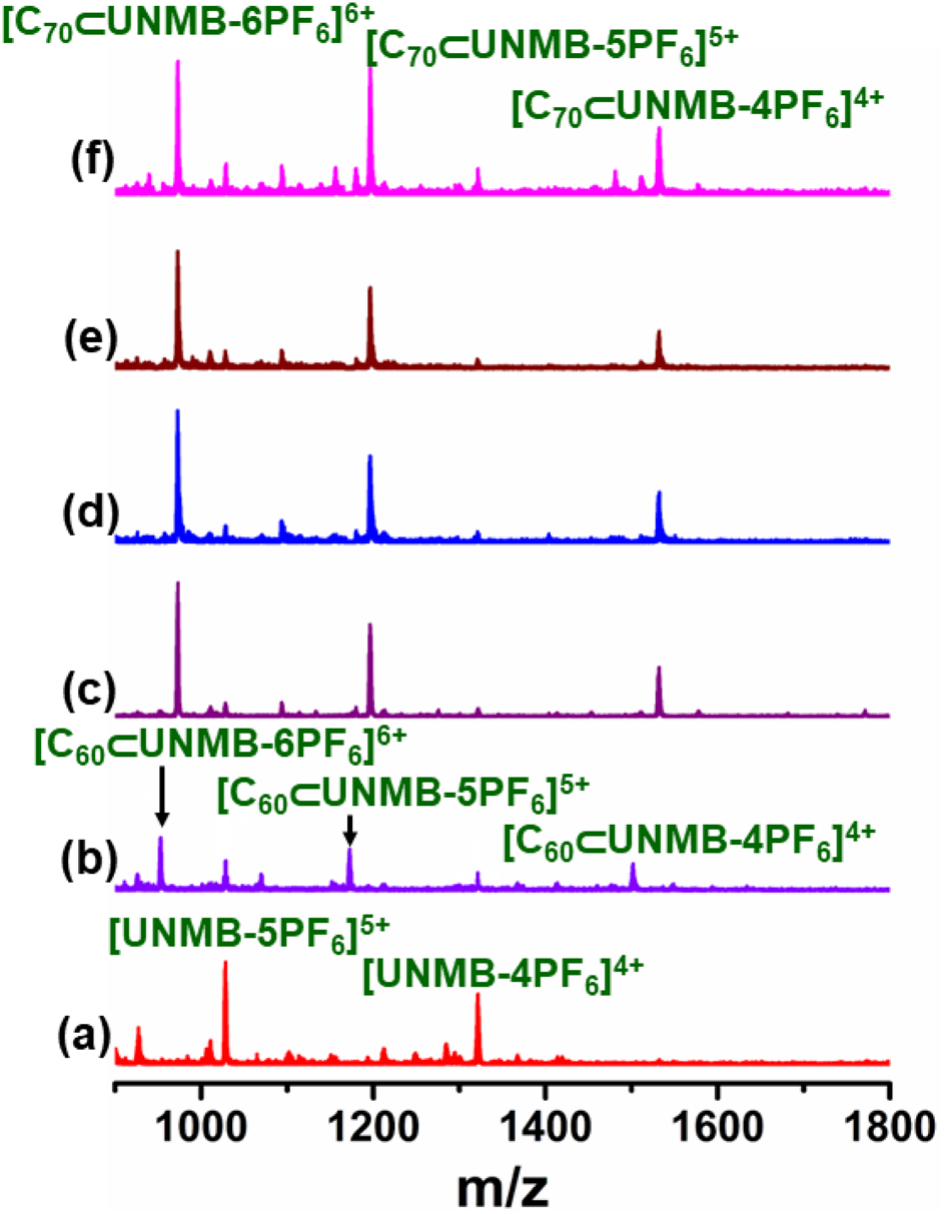
Partial ESI-MS spectra of (a) UNMB, (b) C_60_⊂UNMB, (c) C_70_⊂UNMB, and (d) UNMB treated with an equimolar (2 eqv. each) mixture of C_60_ and C_70_, after (e) treatment of C_70_⊂UNMB with C_60_, and after (f) treatment of C_70_ with C_60_⊂UNMB.

The stronger binding ability of UNMB for C_70_ over C_60_ suggested by ESI-MS and the association constant values is an exciting finding, which gave us the idea to examine the selective binding ability of UNMB for the selective extraction of one fullerene from a mixture of C_60_/C_70_. Such extraction is very challenging due to the poor solubility of fullerenes in common solvents. To do this, first, a competitive inclusion experiment was performed. To an acetonitrile solution of UNMB, an equimolar mixture of C_60_ and C_70_ (2 equiv. of each) was added, and the mixture was heated at 55 °C for 12 h under stirring. The resulting deep purple supernatant was analyzed by ESI-MS, which exhibited the spectral features of C_70_⊂UNMB (Fig. 8d). Furthermore, an acetonitrile solution of C_70_⊂UNMB was treated with 2 equivalents of solid C_60_ at 55 °C for 12 h. The ESI-MS spectrum of the so-obtained deep purple solution showed the characteristic pattern of C_70_⊂UNMB (Fig. 8e). Next, we did this experiment in the reverse manner, and 2 equivalents of solid C_70_ were added to the violet acetonitrile solution of C_60_⊂UNMB and stirred at 55 °C for 12 h. This again gave a deep purple solution that showed the ESI-MS spectral data of C_70_⊂UNMB (Fig. 8f). Thus, the C_70_ introduced into the C_60_⊂UNMB solution replaces the bound C_60_, but C_60_ could not substitute the bound C_70_ from the cavity of UNMB. Therefore, above experiments also confirm that UNMB has a stronger binding tendency towards C_70_ than C_60_.

**Scheme 2:**
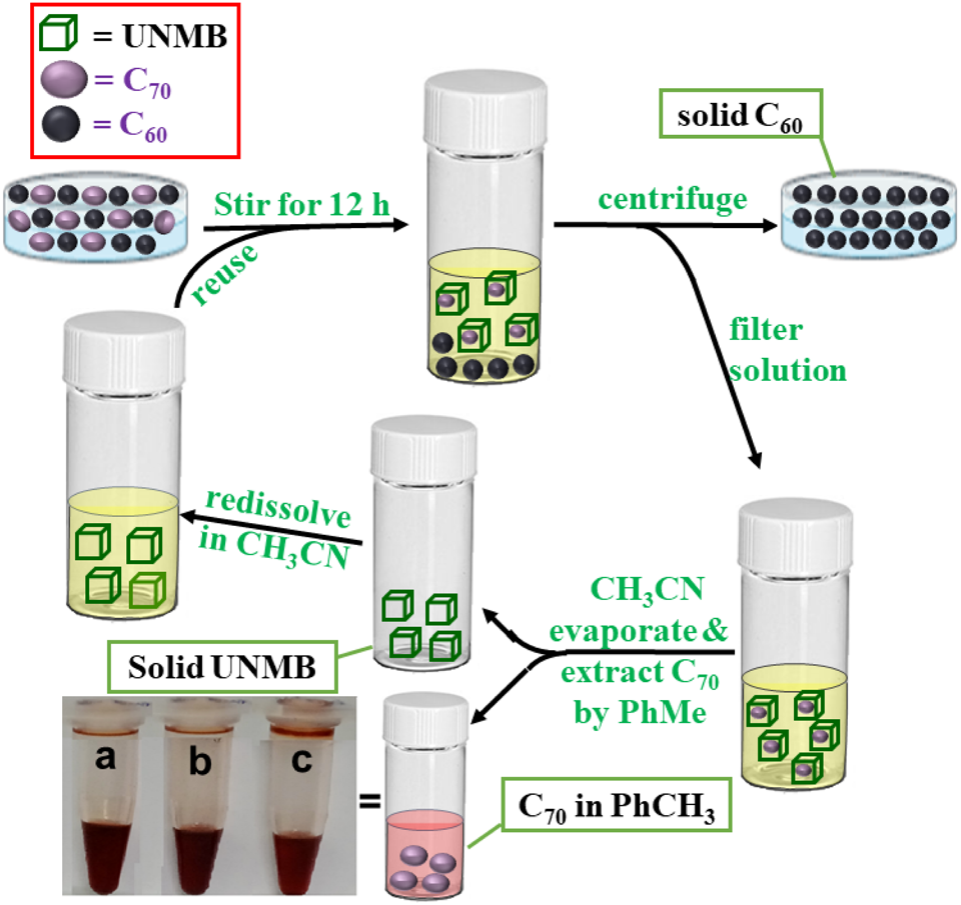
Schematic presentation of the separation of fullerene C_70_ from a mixture of C_60_ and C_70_ by UNMB. (Inset picture shows a, b and c labelled vials containing toluene solutions of extracted C_70_ after the 1st, 2nd and 3rd cycles, respectively).

To investigate whether UNMB has the overall ability to extract C_70_ from a C_70_/C_60_ mixture, an equimolar mixture of C_70_/C_60_ was treated with 1 mL (10 mg) of an acetonitrile solution of UNMB and heated at 55 °C with stirring for 12 h. The centrifuged acetonitrile solution containing predominately C_70_⊂UNMB was evaporated, and the brown solid thus obtained was treated with 0.5 mL of toluene overnight with stirring at room temperature. The resulting suspension was centrifuged, and the supernatant was investigated by UV-vis analysis. The absorption spectrum of the supernatant in toluene exhibited the absorption profile corresponding to C_70_ (Fig. S29†). Thus, the UNMB barrel is found to be a potential receptor for the separation of fullerene C_70_ from its homologue C_60_ (Scheme 2). This is a very stimulating finding as most of the reported fullerene receptors suffer from strong binding without any selectivity and once the guests are bound, their removal is difficult.

The solid residue in the acetonitrile showed an ESI-MS pattern very similar to that of the as-synthesized UNMB, along with the good isotopic distribution patterns of several charge fragments (Fig. S30†). This is a fascinating observation that prompted us to check the reusability of barrel UNMB as a C_70_ extracting agent. We found that UNMB can be reused for the extraction of C_70_ with high purity for three cycles.

## Conclusions

In conclusion, we have designed and synthesized an unsymmetrical tetratopic ligand that has two different donor groups (pyridine/imidazole). Its self-assembly with a *cis*-Pd(ii) acceptor in a 1 : 2 molar ratio yielded a low symmetry tetrafacial molecular barrel M_8_L^un^_4_ (UNMB). The formation of an isomeric molecular barrel (HHTT) was suggested by NMR studies in solution and by the solid-state single-crystal X-ray structure analysis. UNMB has two large open windows and a large cavity enclosed by aromatic panels from the unsymmetrical ligands. These features make it a suitable host for binding with large guests like C_70_ and C_60_ through noncovalent interactions (π–π interaction) between the aromatic panels of the barrel and the fullerenes. Encapsulation of fullerenes resulted in an increase in the solubility of C_60_/C_70_ in acetonitrile, which otherwise are insoluble in the absence of UNMB. ESI-MS analysis revealed the formation of 1 : 1 host–guest inclusion complexes for C_70_ and C_60_, which was further supported by UV-vis titration experiments. UV-vis titration experiments and competitive guest uptake experiments corroborated that UNMB has stronger binding affinity towards C_70_ over its spherical analogue C_60_, which enabled it to exclusively form a C_70_⊂UNMB inclusion complex from a mixture of C_60_ and C_70_. This preferential binding ability of UNMB for C_70_ over C_60_ was employed to separate C_70_ from a mixture of C_60_/C_70_ with high purity. Moreover, the encapsulated fullerene in pure form was extracted using toluene and the recovered UNMB was reused for C_70_ separation for up to three cycles.

## Data availability

All data (NMR, ESI-MS) are provided in the ESI,† and additional data will be available upon request.

## Author contributions

P. S. M. and D. P. designed the project and devised the experiments. D. P. carried out the experimental work, analysed the data, optimized the structures, and carried out the theoretical calculations. J. K. C. collected and solved the crystallographic data. All the authors contributed to the writing of the manuscript.

## Conflicts of interest

There are no conflicts to declare.

## Supplementary Material

SC-015-D4SC01332H-s001

SC-015-D4SC01332H-s002
