# Supplementary material for: Formation of a low-symmetry Pd8 molecular barrel employing a hetero donor tetradentate ligand, and its use in the binding and extraction of C70
Source: Chem Sci. 2024 Jun 26;15(31):12502–10. doi: 10.1039/d4sc01332h (PMC11304780; doi:10.1039/d4sc01332h)
Supplement: SC-015-D4SC01332H-s001 [file SC-015-D4SC01332H-s001.pdf]

Electronic Supplementary Material (ESI) for Chemical Science.
This journal is © The Royal Society of Chemistry 2024

Supporting Information

Formation of low-symmetric Pd_8 molecular barrel employing a hetero donor tetradentate ligand and its use in binding and extraction of C_{70}

Dharmraj Prajapati[a], Jack K. Clegg[b] and Partha Sarathi Mukherjee[a]*

[a]*Department of Inorganic and Physical Chemistry, Indian Institute of Science, Bangalore-560012, India*

[b]*School of Chemistry and Molecular Biosciences, The University of Queensland, St. Lucia, Queensland 4072, Australia.*

*Email: psm@iisc.ac.in

Materials and Methods:

All the chemicals and solvents were purchased from different commercially available suppliers and directly used without further purification. NMR studies were performed on Bruker 400 and 500 MHz spectrometers. The chemical shifts (δ) are accounted in parts per million (ppm) unit relative to tetramethylsilane (Me_4Si) as an internal standard (0.0 ppm), and proton resonance appeared due to incomplete deuteration of the solvents $CDCl_3$ (7.26 ppm), $(CD_3)_2SO$ (2.50 ppm) and D_2O (4.79 ppm). ^{13}C NMR spectra were collected on the same instruments at 100 MHz, 125 MHz, and chemical shift (δ) were assigned in ppm units relative to external $CDCl_3$ at 77.4–76.8 ppm. Electrospray ionization mass spectrometry (ESI-MS) experiments were recorded on an Agilent 6538 Q-TOF ESI-MS instrument using spectroscopic grade solvents. All the UV-Vis studies were carried out on a PerkinElmer Lambda-750 spectrophotometer.

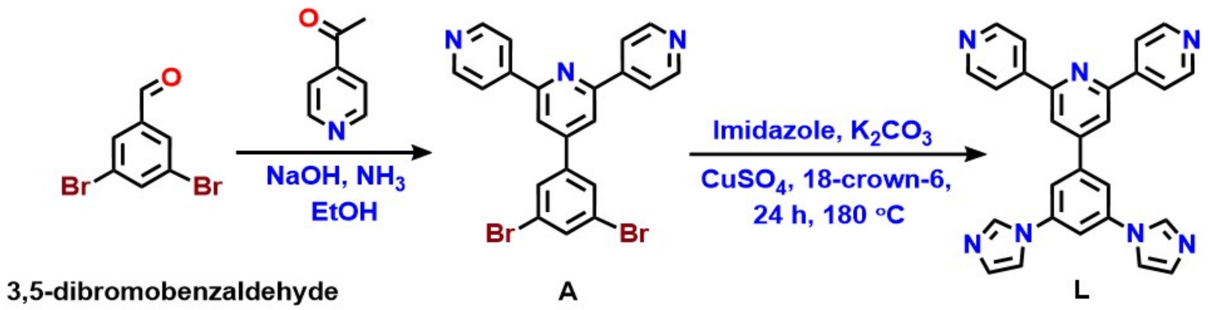

Scheme S1: Synthetic route of the ligand **L^un^**.

Synthesis of 4'-(3,5-dibromophenyl)-4,2':6',4''-terpyridine (A):

The 3,5-dibromobenzaldehyde (1000 mg, 3.79 mmol) was added to a 250 mL round bottom flask containing 50 mL of ethanol at 0 ºC followed by the addition of NaOH (181.88 mg, 4.54 mmol) while stirring. To this cold solution 4-acetyl pyridine (1.01 g, 8.34 mmol) in 10 mL ethanol was added dropwise over 10 min. Finally, ammonia solution (20 mL, 30% weight) was added to the solution and kept for stirring at 0 ºC for 1 h. The resulted solution was allowed to reach to room temperature and refluxed at 80 for 24 h. Magenta precipitate was formed, which was filtered and washed with cold ethanol, water, and ethanol to isolate pure product as a soft pink solid (760 mg, 43% yield). ^{1}H NMR (500 MHz; DMSO-d_6) δ (ppm): 8.77 (d, 4H), 8.56 (s, 2H), 8.42 (s, 2H), 8.36 (d, 4H), 8.01 (s, 2H).

Synthesis of 4'-(3,5-di(*1H*-imidazol-1-yl) phenyl)-4,2':6',4''-terpyridine (L^{un}):

The ligand L was prepared by Ullmann coupling reaction following the literature procedure.[1] Compound A (750 mg, 1.57 mmol), imidazole (427 mg, 6.28 mmol), K_2CO_3 (866.64 g, 6.28 mmol), and $CuSO_4$ (0.01 g, 0.06 mmol) were mixed in a 50 mL flask and heated under a nitrogen atmosphere for 24 h to 185 °C. The reaction mixture was then cooled to ambient temperature and was washed three times with water. The remaining solid residue was extracted with methanol (70 mL). The methanol solution was concentrated in volume and desired ligand **L^{un}** was precipitated out as a white solid from methanol concentrated solution upon addition of excess of distilled water. The white precipitate was filtered and washed with water and brought to dryness to give a colourless solid. Yield: 43% (300 mg, 0.68 mmol). ^{1}H NMR (500 MHz; DMSO-d_6) δ (ppm): 8.82 (d, 4H), 8.10 (d, 4H), 8.53 (s, 2H), 8.02 (s, 2H), 7.73 (s, 2H), 7.53 (s, 1H), 7.43 (s, 2H), 7.32 (s, 2H).

Synthesis of Pd_8L_4 Molecular Barrel (UNMB):

The solid terpyridine-diimidazole ligand $\mathbf{L^{un}}$ (100 mg, 0.23 mmol) was added to a solution of *cis*-(tmeda)Pd(NO_3)$_2$ (M1) (157 mg, 0.45 mmol) in 1.5 mL DMSO and the resulting mixture was heated at 60 °C with stirring for 12 h. The reaction mixture turned into a clear pale-yellow solution, which was centrifuged, and the filtrate was then treated with excess of ethyl acetate to obtain white product as precipitate. The product was collected by centrifugation, washed with acetone, ether, and dried in vacuum. Isolated yield: 95 % (250 mg, 0.055 mmol). ^{1}H NMR (400 MHz, D_2O): δ = 9.18 (m, 4H), 8.94 (s, 2H), 8.50–8.35 (m, 4H), 8.32–8.30 (m, 2H), 8.18–8.04 (m, 2H), 7.81 (s, 1H), 7.71 (s, 2H), 7.67–7.58 (m, 2H), 3.13–3.22 (m, 8H), 2.91–2.70 (m, 24H). ^{13}C NMR (100 MHz, CD_3CN): δ = 152.91, 152.13, 150.82, 150.75, 149.44, 140.83, 138.37, 137.78, 129.66, 129.52, 125.63, 124.73, 122.20, 121.91, 121.24, 120.34, 118.42, 115.98, 63.35, 63.23, 62.98, 62.87, 62.47, 51.58, 51.32, 51.16, 51.05, 50.90, 50.77. ESI-MS: (CH_3CN): m/z = 1810.4826 (calcd 1810.4936) for $[M_8L^{un}{}_4(PF_6)_{13}]^{3+}$, 1321.6334 (calcd 1321.6366) for $[M_8L^{un}{}_4(PF_6)_{12}]^{4+}$, 1028.3242 (calcd 1028.3165) for $[M_8L^{un}{}_4(PF_6)_{11}]^{5+}$, 832.7648 (calcd 832.7697) for $[M_8L^{un}{}_4(PF_6)_{10}]^{6+}$, 693.0971 (calcd 693.0934) for $[M_8L^{un}{}_4(PF_6)_9]^{7+}$.

C_{70} Encapsulation:

A clear solution of **UNMB** (10.0 mg, 0.0017 mmol) in acetonitrile (0.5 mL) was treated with 2 equivalents of C_{70} (2.86 mg, 0.0034 mmol) and stirred at 55°C for overnight. The resulting solution turned into violet, and it was centrifuged to remove excess fullerene C_{70}. Clear solution was then treated with excess diethyl ether which yielded **UNMB⊃C_{70}** almost in quantitative yield. Yield: 99%. ^{1}H NMR (400 MHz, CD_3CN): δ = 9.09 (d, 2H), 8.98(s), 8.73 (d), 8.38 (s), 8.25(s), 8.13 (s), 8.10 (s), 7.87 (d), 7.76 (s), 7.72 (s), 7.57(s). 3.04–2.97 (br, 8H), 2.77–2.62(br, 24H)^{13}C NMR (125 MHz, CD_3CN): δ = 150.79, 149.95, 146.76, 146.59,146.15, 143.87, 138.32, 137.78, 129.92, 129.67, 121.05, 120.33, 63.35, 63.28, 62.88, 62.48, 51.33, 51.16, 50.78. ESI-MS: (CH_3CN): m/z = 1531.6380 (calcd 1531.6366) for $[C70{\subset}M_8L^{un}{}_4(PF_6)_{12}]^{4+}$, 1196.3206 (calcd 1196.3165) for $[C70{\subset}M_8L^{un}{}_4(PF_6)_{11}]^{5+}$, 972.7755 (calcd 972.7697) for $[C70{\subset}M_8L^{un}{}_4(PF_6)_{10}]^{6+}$, 813.0999 (calcd 813.0934) for $[C70{\subset}M_8L^{un}{}_4(PF_6)_9]^{7+}$, 693.3494 (calcd 693.3362) for $[C70{\subset}M_8L^{un}{}_4(PF_6)_8]^{8+}$.

C_{60} Encapsulation:

Host-guest inclusion complex **C_{60}⊂UNMB** was synthesized following the same procedure as used for C_{70}⊂**UNMB**. Barrel **UNMB** (10.0 mg, 0.0017 mmol) and C_{60} (2.45 mg, 0.0034 mmol) were used as starting materials. After completion of the reaction, the solution colour changed from colourless to deep purple. Yield 98%. ^{1}H NMR (400 MHz, CD_3CN): δ = 9.06 (d, 4H), 8.90 (s, 2H), 8.72 (d, 2H), 8.48 (d, 2H), 8.39 (s, 1H), 8.26 (d, 2H), 8.25 (s, 1H), 8.13 (s, 1H), 7.87 (s, 1H), 7.77 (s, 1H), 7.73 (s, 1H), 7.57 (d, 1H), 3.03–3.02 (br 8H), 2.77–2.64 (br 24H). ^{13}C NMR (500 MHz, CD_3CN): δ = 152.90, 152.80, 152.48, 150.92, 150.76, 150.68, 147.02, 141.99, 140.76, 138.37, 137.82, 129.75, 129.59, 126.60, 125.85, 124.69, 122.15, 121.81, 121.02, 63.27, 63.18, 51.51, 51.26, 51.08, 50.99, 50.88, 50.69, 50.61. ESI-MS: (CH_3CN): m/z = 1501.6285 (calcd 1501.6366) for $[C60{\subset}M_8L^{un}{}_4(PF_6)_{12}]^{4+}$, 1172.3069 (calcd 1172.3165) for $[C60{\subset}M_8L^{un}{}_4(PF_6)_{11}]^{5+}$, 952.7668 (calcd 952.7697) for $[C60{\subset}M_8L^{un}{}_4(PF_6)_{10}]^{6+}$, 795.9456 (calcd 795.9506) for $[C60{\subset}M_8L^{un}{}_4(PF_6)_9]^{7+}$, 678.3319 (calcd 678.3362) for $[C60{\subset}M_8L^{un}{}_4(PF_6)_8]^{8+}$.

Theoretical Calculations:

All the theoretical calculations were performed using Gaussian 09 package.[2] All the isomers HHHH, HHHT, HHTT, HTHT of **UNMB**, **C_{70}⊂UNMB**, and **C_{60}⊂UNMB** were optimized using PM6 semi-empirical method. No symmetry constraints were used during the optimization procedure. Single point energies to compare their relative stabilities of the isomers HHHH, HHHT, HHTT and HTHT and C_{70}/C_{60}⊂UNMB were calculated using the B3LYP functional with mixed basis set of LanL2DZ (for Pd atom) and 631-G (for C, H, and N atoms).

Determination of Association Constant:

The association constant (K_a) for **C_{70}⊂UNMB** and **C_{60}⊂UNMB** formation was determined by UV-visible titration in acetonitrile. The concentrated solution of C_{70} (1 mM in toluene) or C_{60} (1 mM in toluene) was added incrementally to the solution of 1 (10 μM in acetonitrile) and UV-Vis spectra were recorded one after the other. From the UV-Vis titration experiment, plots (Figure S11, S13) of absorption intensity at 306 nm against the equiv. of guests (C_{70} or C_{60}) were obtained and a nonlinear least squares data treatment[2] gave association constants of 7.15 × 10^5 M^{-1} for **C_{70}⊂UNMB** and 2.83 × 10^4 M^{-1} for **C_{60}⊂UNMB**.

Selective Extraction of C_{70} from C_{60}:

To a 1 mL acetonitrile (10 mg, 0.0017 mmol) colourless solution of **UNMB** in a 4 mL glass vial, 2.86 mg (0.034 mmol) of C_{70}, and 2.34 mg (0.0034 mmol) of C_{60} were added. The resulting suspension was stirred for 12 hours at 55° C. After completion of the reaction, resulting suspension was centrifuged to remove unencapsulated guest. The supernatant containing host-guest complex was treated with toluene to extract guest molecules. UV-vis analysis of the extracted guest in toluene exhibited the absorption pattern of C_{70} only. Acetonitrile solution of **UNMB** after extraction of the guest showed an almost identical ^{1}H NMR and ESI-MS data to that of the as synthesized **UNMB**.

Single Crystal Data Collection and Structure Solution of UNMB:

Single-crystal X-ray data for **UNMB** were collected using an Oxford Rigaku Synergy-S employing confocal mirror monochromated Mo-K_{α} radiation generated from a microfocus source (0.71073 Å) with ω and ψ scans at 150(2) K.[1] Data integration and reduction were undertaken with CrysAlisPro[1]. Subsequent computations were carried out using Olex2.[2] Structures were solved with ShelXT[3] and refined and extended with ShelXL.[4] Non-hydrogen atoms were refined anisotropically, carbon-bound hydrogen atoms were included in idealised positions and refined using a riding model. The structure has a large volume of smeared electron density in the lattice corresponding to highly disordered solvents (water) and anions (nitrate) which could not be successfully modelled. This region of electron density was therefore treated with the solvent masking[5] algorithm of Olex2. Two of the tetramethylethylenediamine ligands (those coordinated to Pd(5) and Pd(8) have relatively large thermal parameters which arises from unresolved disorder in these groups, which consequently results in the less that ideal placement of hydrogen atoms resulting in short hydrogen-hydrogen contacts which gives rise of several checkcif alerts. The connectivity, however, is unambiguous. Crystallographic data and refinement parameter are given below. The CIF has been deposited with the CCDC number 2300816.

Crystal Data for $C_{156}H_{266}N_{60}O_{80}Pd_{8}$ (M =5113.47 g/mol): triclinic, space group P-1 (no. 2), a = 21.8333(4) Å, b = 21.8629(3) Å, c = 30.2029(6) Å, α = 87.8618(14)°, β = 77.0258(15)°, γ = 82.1629(14)°, V = 13917.4(4) Å^3, Z = 2, T = 150(2) K, μ(Mo Kα) = 0.584 mm^{-1}, $Dcalc$ = 1.220 g/cm^3, 269222 reflections measured (3.802° ≤ 2Θ ≤ 50.246°), 49608 unique (R_{int} = 0.0876, R_{sigma} = 0.0591) which were used in all calculations. The final R_1 was 0.0979 (I > 2σ(I)) and wR_2 was 0.3153 (all data). $_{156}H_{266}N_{60}O_{80}Pd_{8}$ (M =5113.47 g/mol): triclinic, space group

P-1 (no. 2), a = 21.8333(4) Å, b = 21.8629(3) Å, c = 30.2029(6) Å, α = 87.8618(14)°, β = 77.0258(15)°, γ = 82.1629(14)°, V = 13917.4(4) Å^3, Z = 2, T = 150(2) K, μ(Mo Kα) = 0.584 mm^{-1}, $Dcalc$ = 1.220 g/cm^3, 269222 reflections measured (3.802° ≤ 2Θ ≤ 50.246°), 49608 unique (R_{int} = 0.0876, R_{sigma} = 0.0591) which were used in all calculations. The final R_1 was 0.0979 (I > 2σ(I)) and wR_2 was 0.3153 (all data).

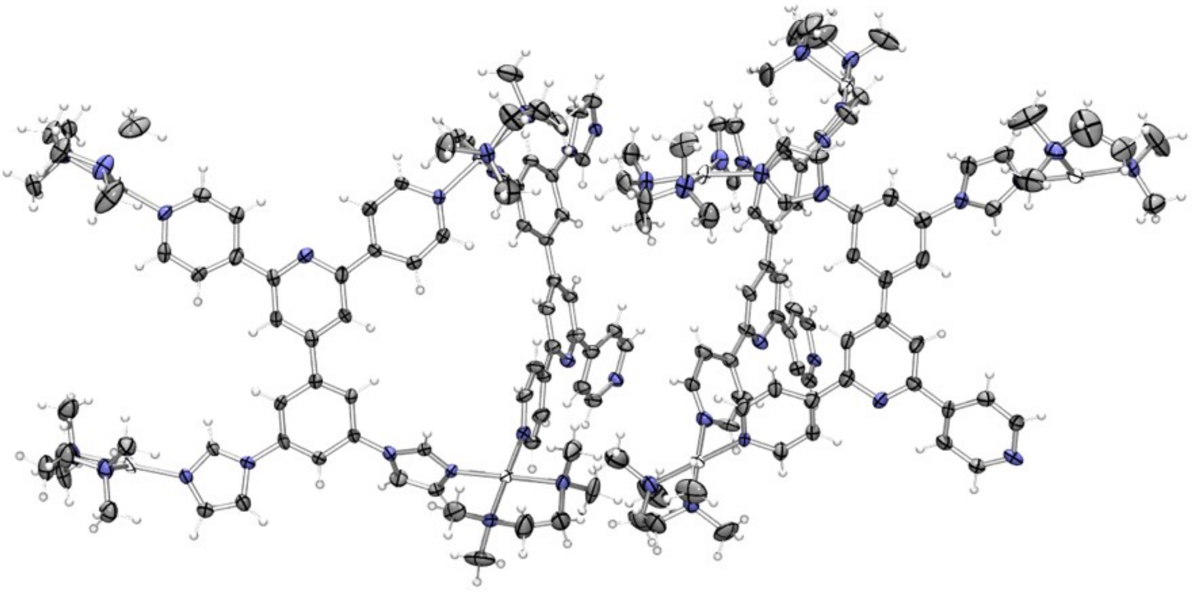

Fig. S1 ORTEP representation of the asymmetric unit of the crystal structure shown with 30 % probability ellipsoids.

Table S1: **Relative single point energy for different diastereomers of UNMB.**

**Solvation**	**HHTT (kcal/mol)**	**HHHT (kcal/mol)**	**HHHH (kcal/mol)**	**HTHT (kcal/mol)**
**Gas Phase**	8.784414	7.198086	0	16.68593
**Water**	12.55131	10.76042	0	24.62572
**DMSO**	12.77972	11.05534	0	24.84786

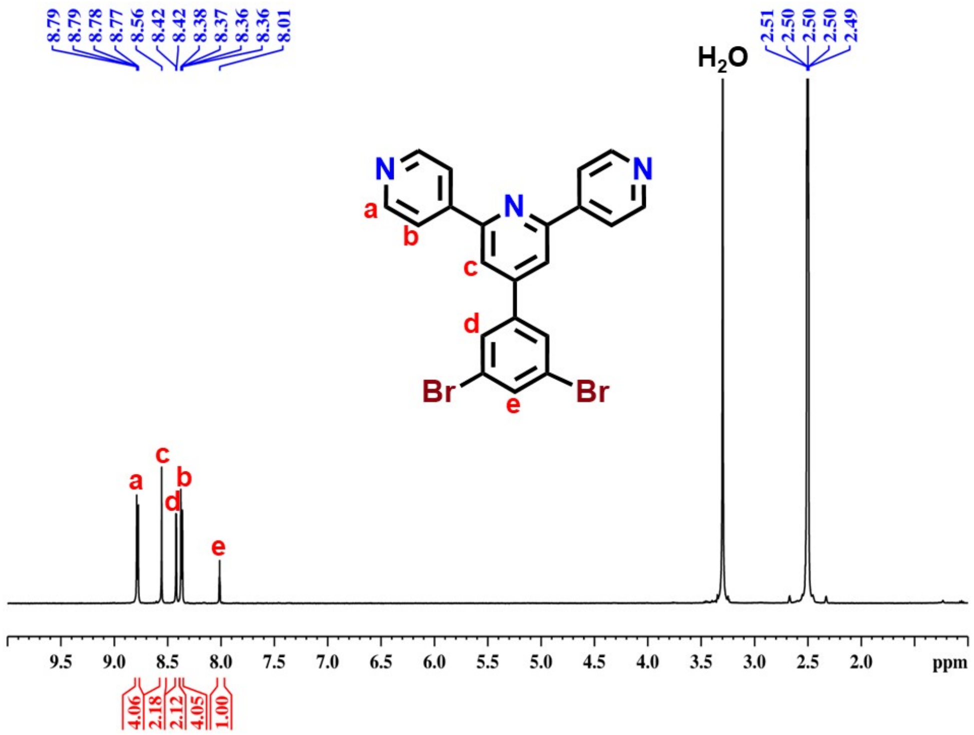

Fig. S2 ^{1}H NMR spectrum of **A** recorded in DMSO-d_6 (500 MHz, 298 K).

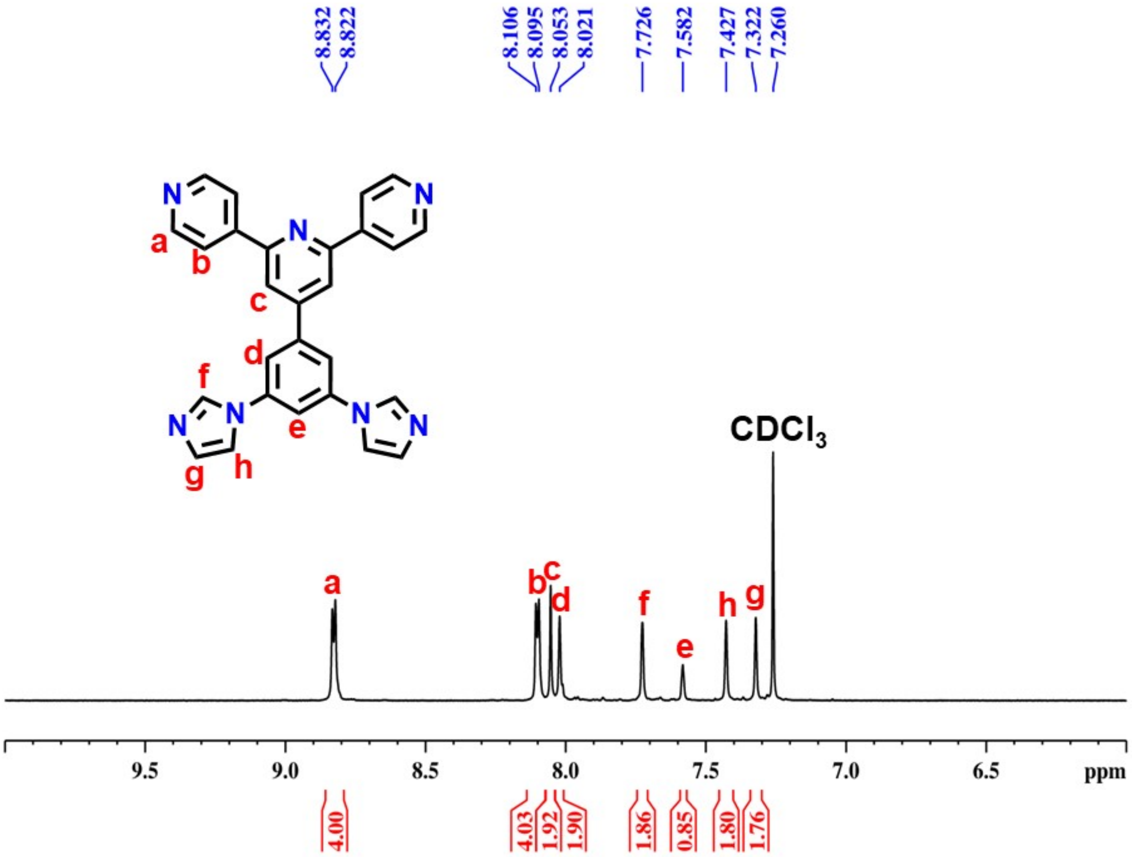

Fig. S3 ^{1}H NMR spectrum of $\mathbf{L^{un}}$ recorded in $CDCl_3$ (500 MHz, 298 K).

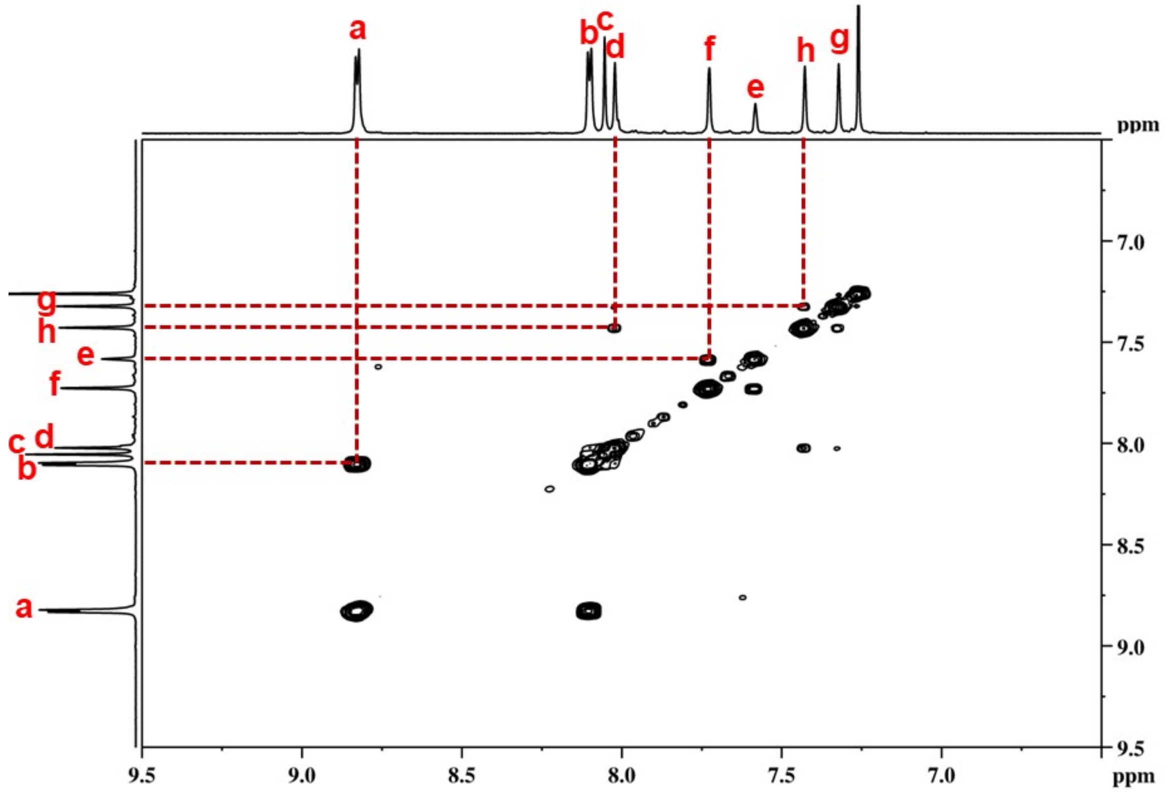

Fig. S4 ^{1}H-^{1}H COSY NMR spectrum of $\mathbf{L^{un}}$ recorded in $CDCl_3$ (500 MHz, 298 K).

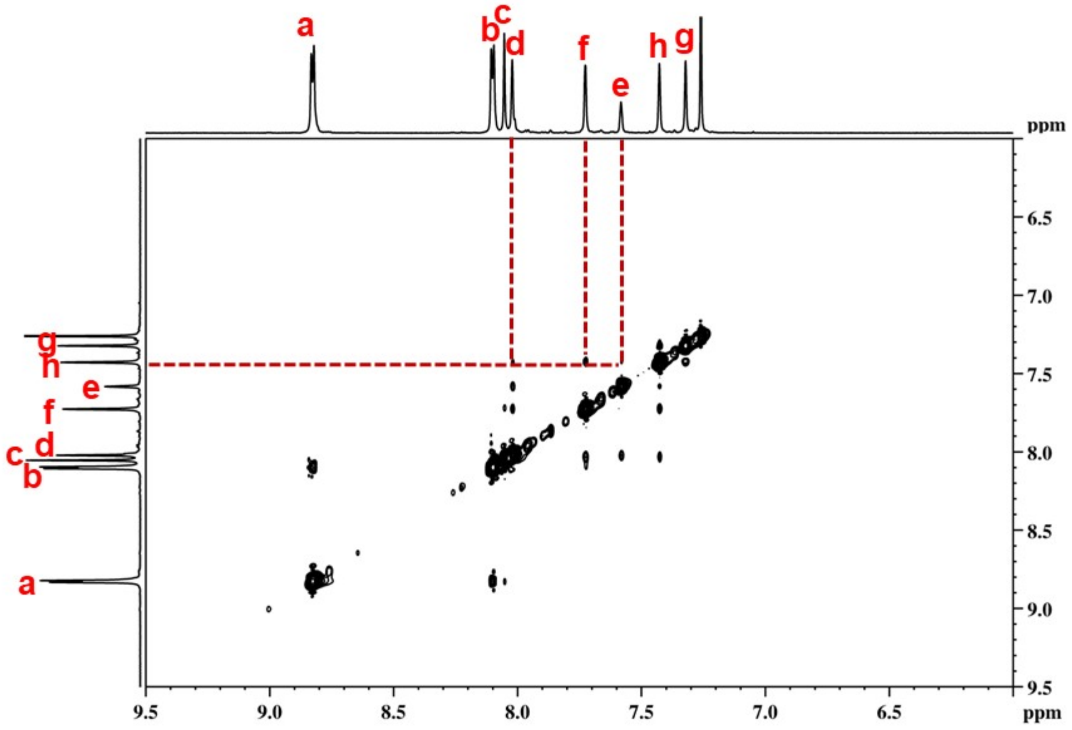

Fig. S5 ^{1}H-^{1}H NOESY NMR spectrum of $\mathbf{L^{un}}$ recorded in $CDCl_3$ (500 MHz, 298 K).

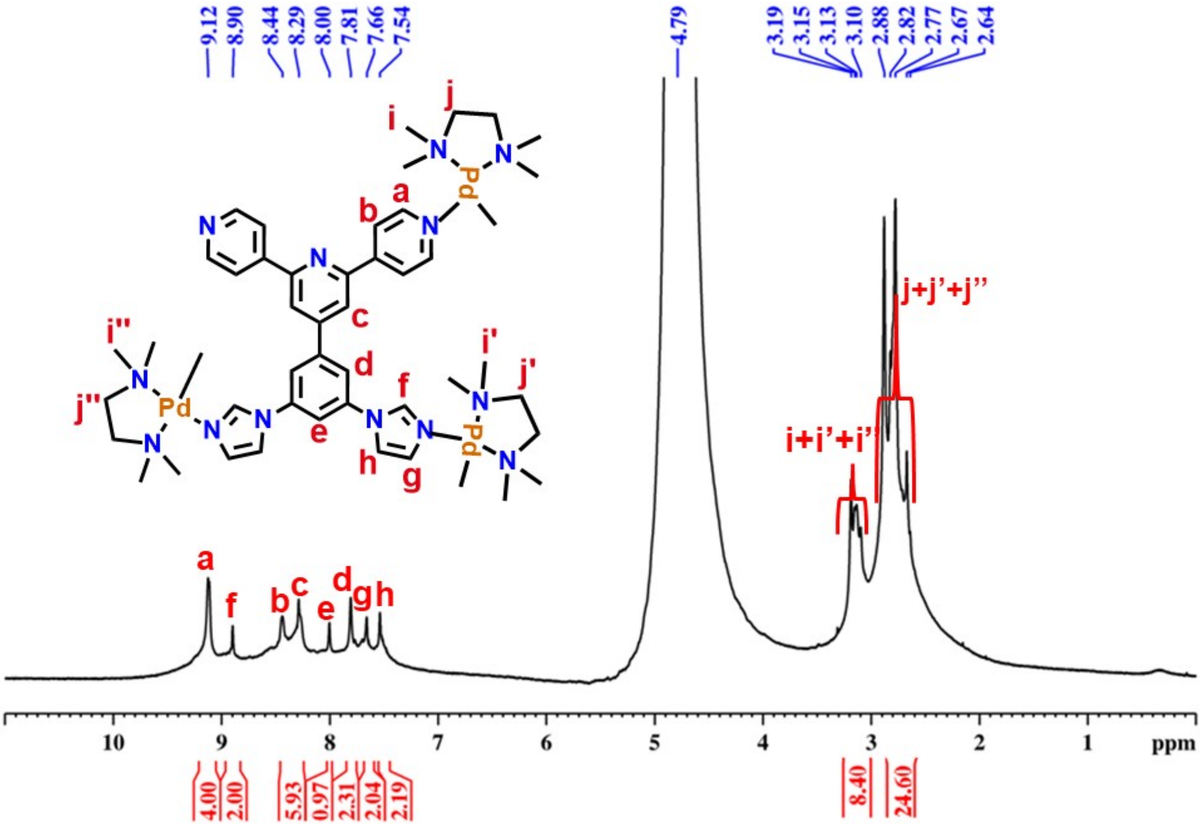

Fig. S6 ^{1}H NMR spectrum of **UNMB** recorded in D_2O (400 MHz, 298 K).

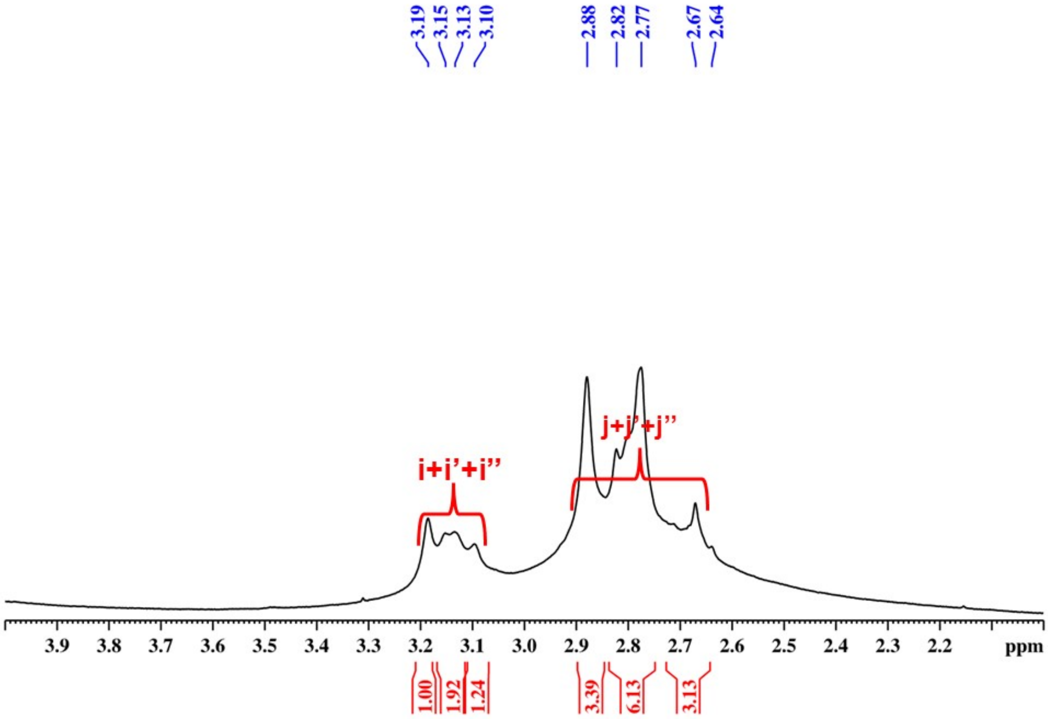

Fig. S7 Partial ^{1}H NMR spectrum of aliphatic region of molecular **UNMB** recorded in D_2O (400 MHz, 298 K).

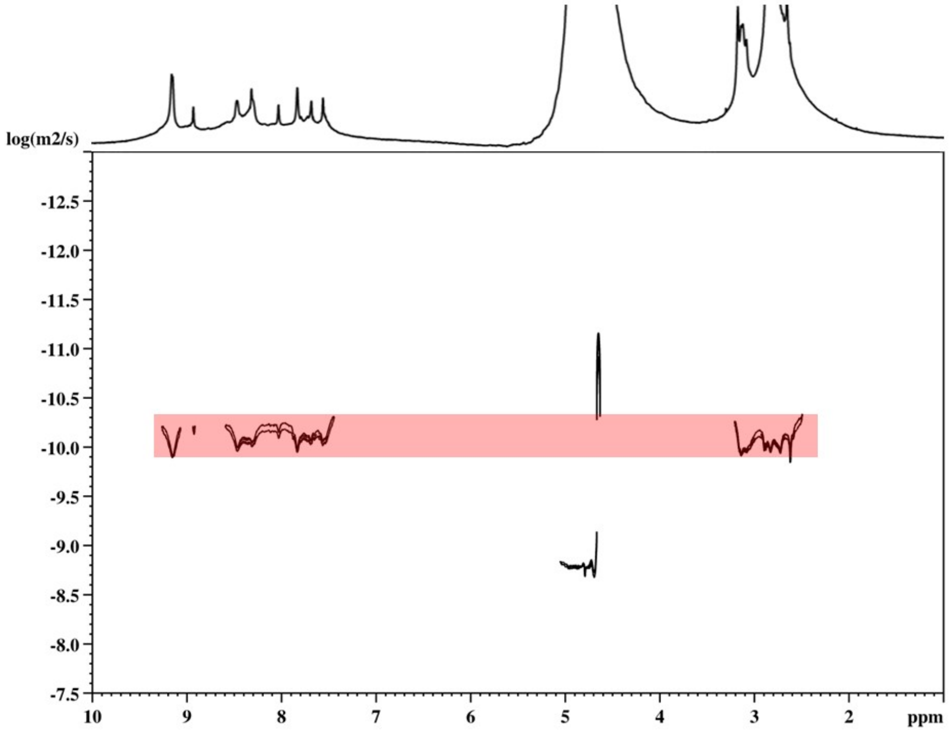

Fig. S8 2D DOSY NMR spectrum of **UNMB** recorded in D_2O (400 MHz, 298 K).

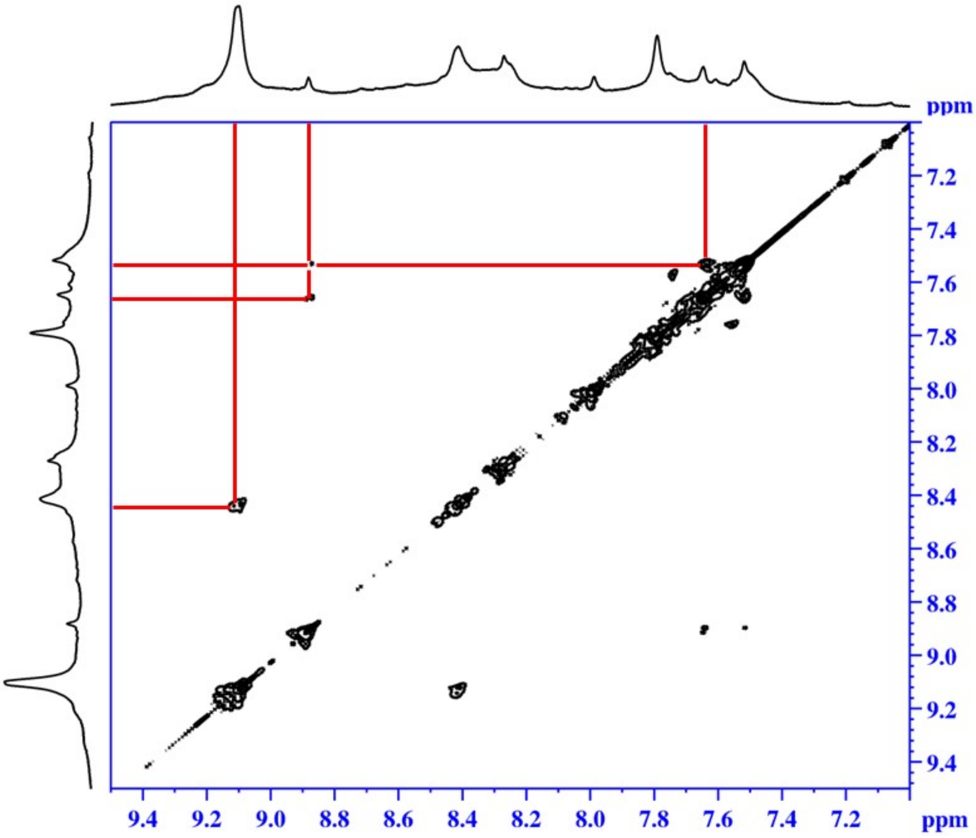

Fig. S9 ^{1}H-^{1}H COSY NMR spectrum of **UNMB** recorded in D_2O (400 MHz, 298 K).

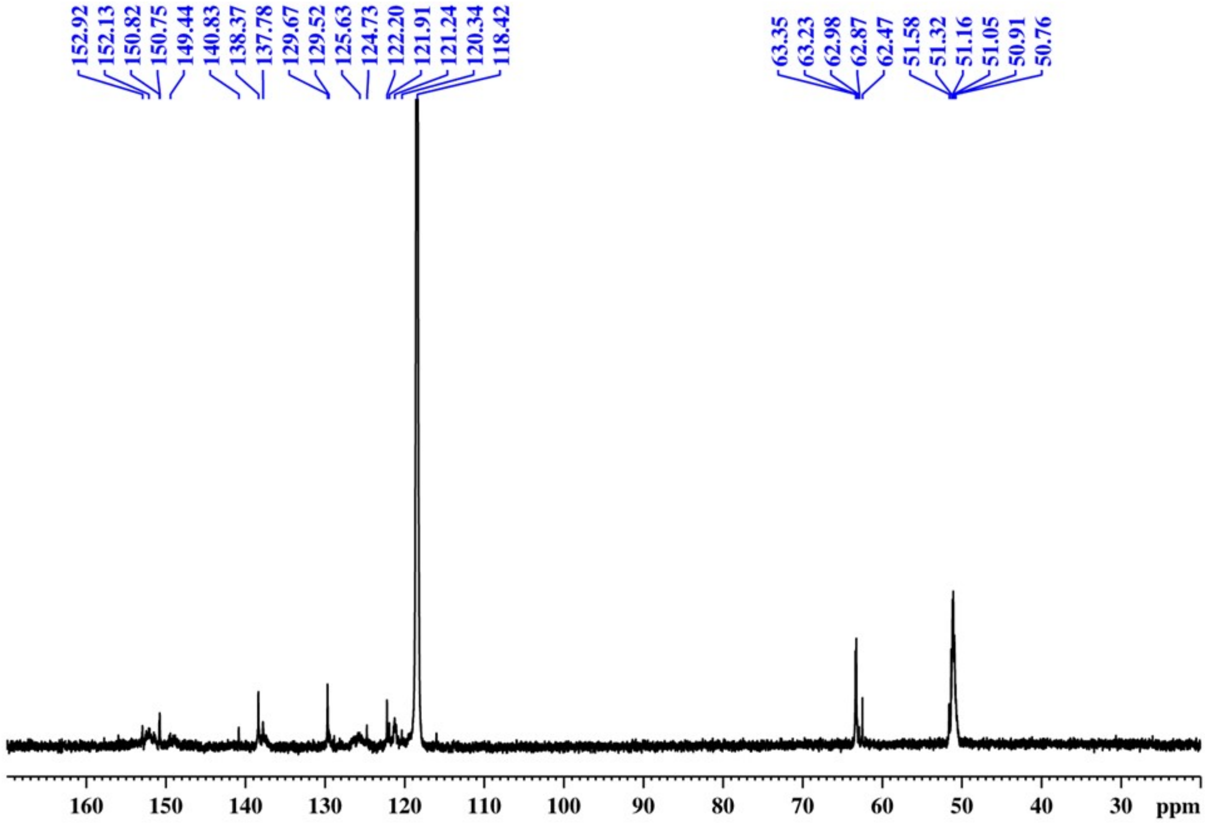

Fig. S10 ^{13}C NMR spectrum of **UNMB** recorded in CD_3CN (500 MHz, 298 K).

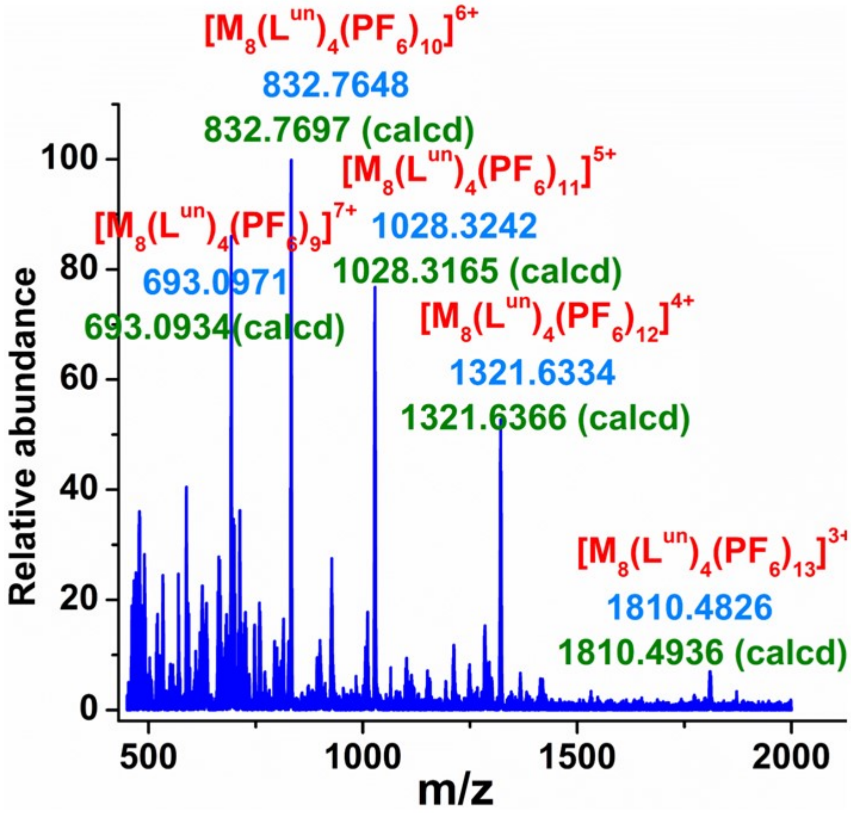

Fig. S11 Full electrospray ionization mass spectrum of **UNMB** recorded in CH_3CN.

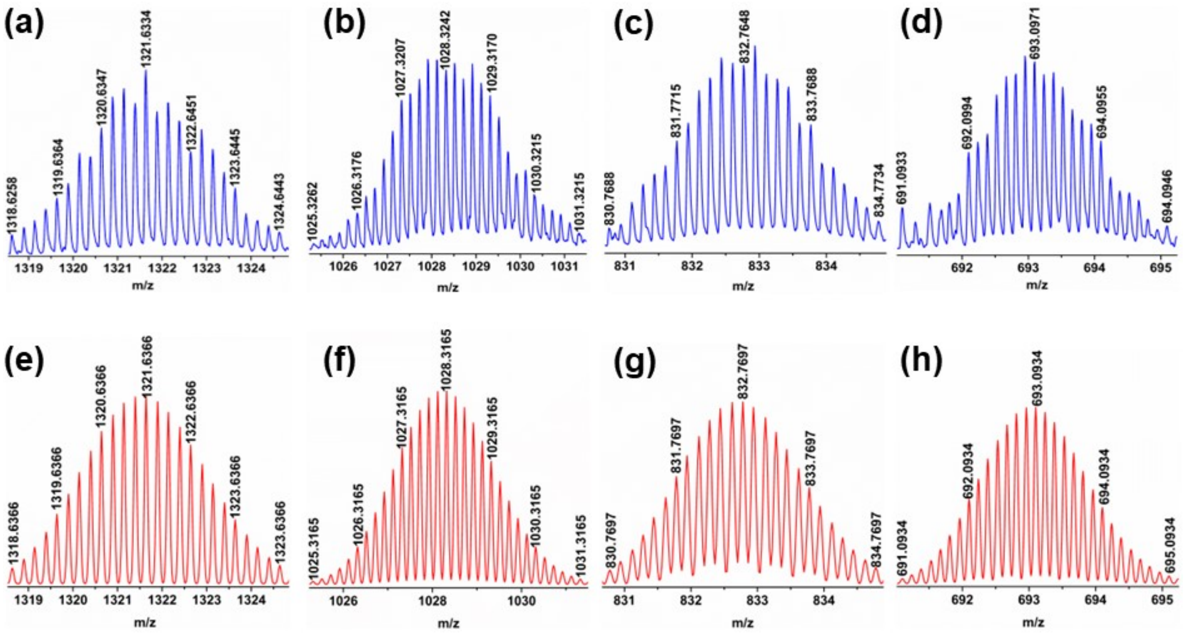

Fig. S12 Experimental (blue) isotopic distribution patterns of the mass-peaks corresponding to $[M_8L^{un}{}_4(PF_6)_{12}]^{4+}$ (a), $[M_8L^{un}{}_4(PF_6)_{11}]^{5+}$ (b), $[M_8L^{un}{}_4(PF_6)_{10}]^{6+}$ (c), and $[M_8L^{un}{}_4(PF_6)_9]^{7+}$ (d). Calculated (red) isotopic distribution patterns of the peaks corresponding to $[M_8L^{un}{}_4(PF_6)_{12}]^{4+}$ (e), $[M_8L^{un}{}_4(PF_6)_{11}]^{5+}$ (f), $[M_8L^{un}{}_4(PF_6)_{10}]^{6+}$ (g), and $[M_8L^{un}{}_4(PF_6)_9]^{7+}$ (h).

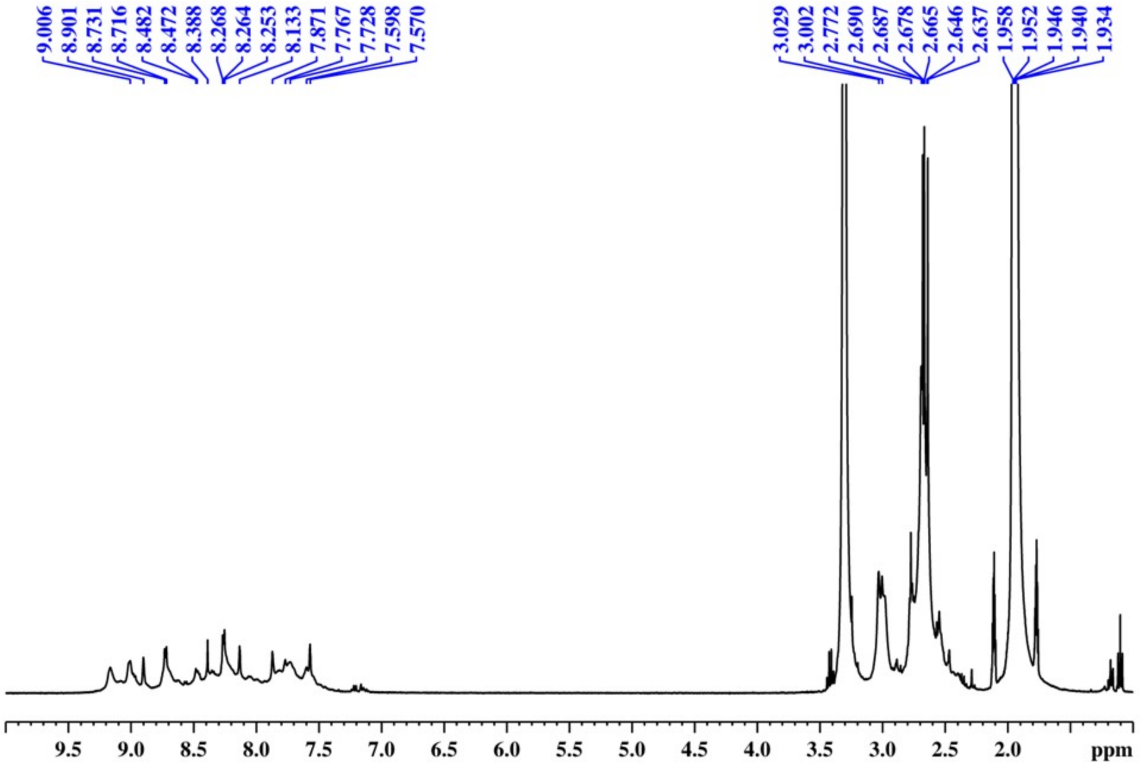

Fig. S13 ^1H NMR spectrum of $\mathbf{C_{60}{\subset}UNMB}$ recorded in CD_3CN (500 MHz, 298 K).

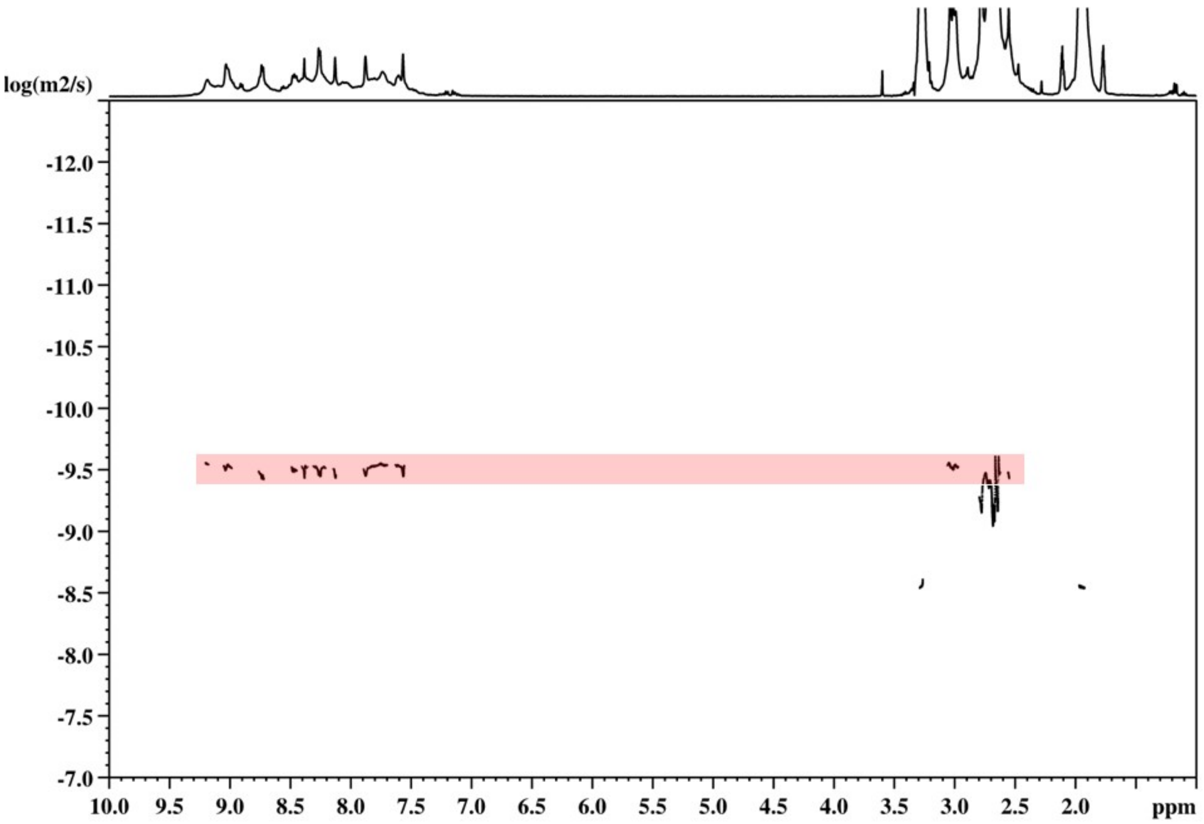

Fig. S14 2D DOSY NMR spectrum of **C_{60}⊂UNMB** recorded in CD_3CN (400 MHz, 298 K).

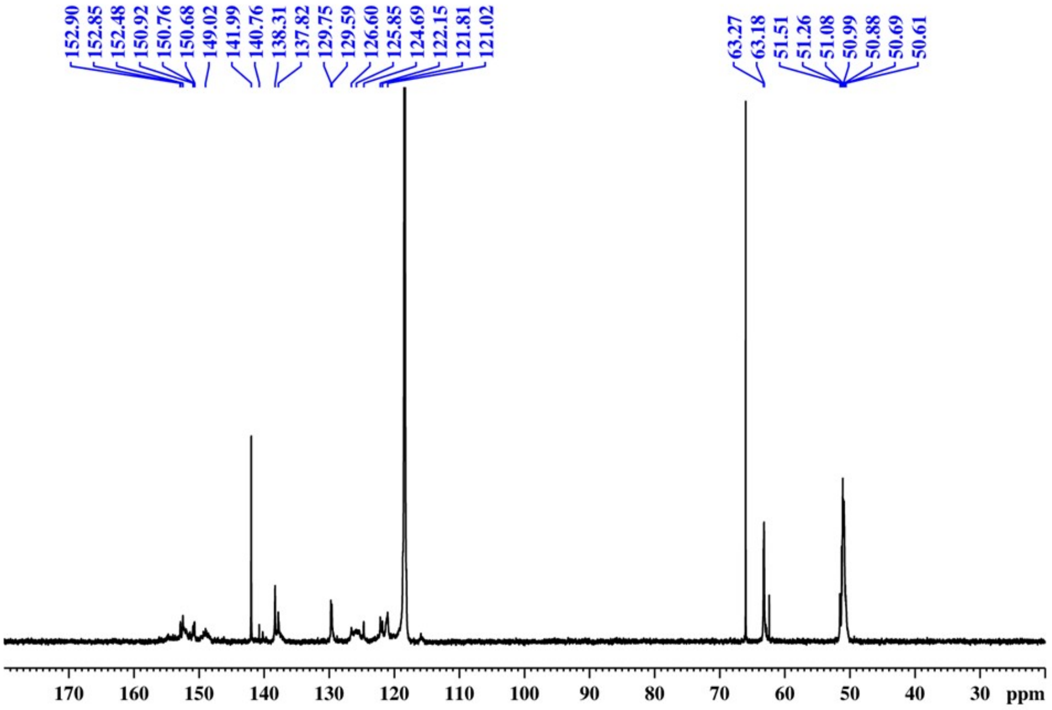

Fig. S15 ^{13}C NMR spectrum of **C_{60}⊂UNMB** recorded in CD_3CN (500 MHz, 298 K).

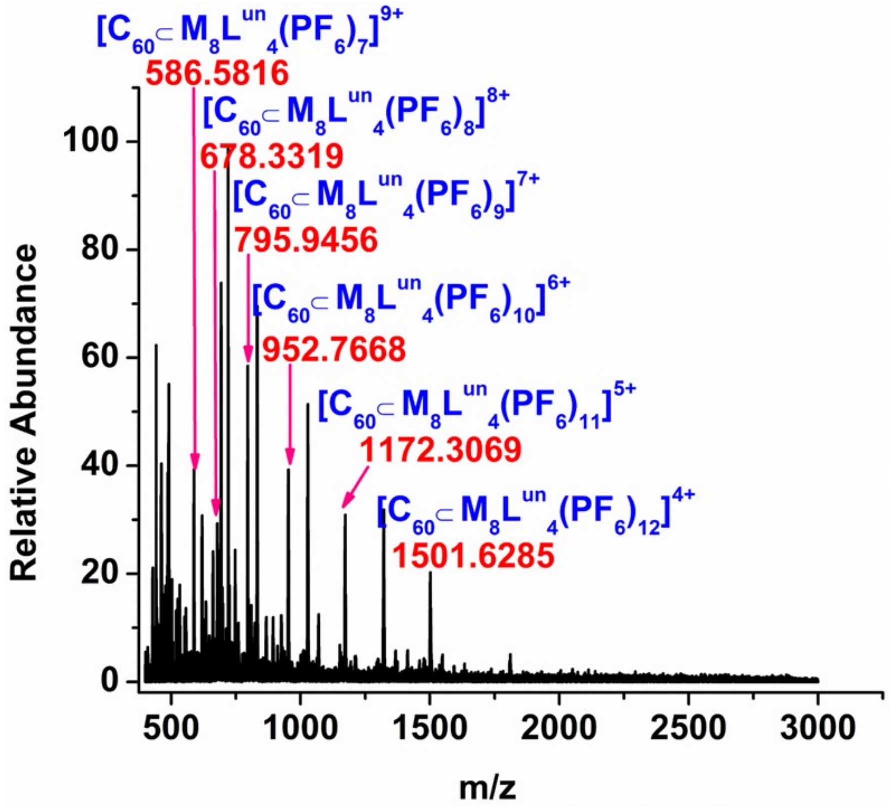

Fig. S16 ESI-MS spectrum of **C_{60}⊂UNMB** recorded in CH_3CN.

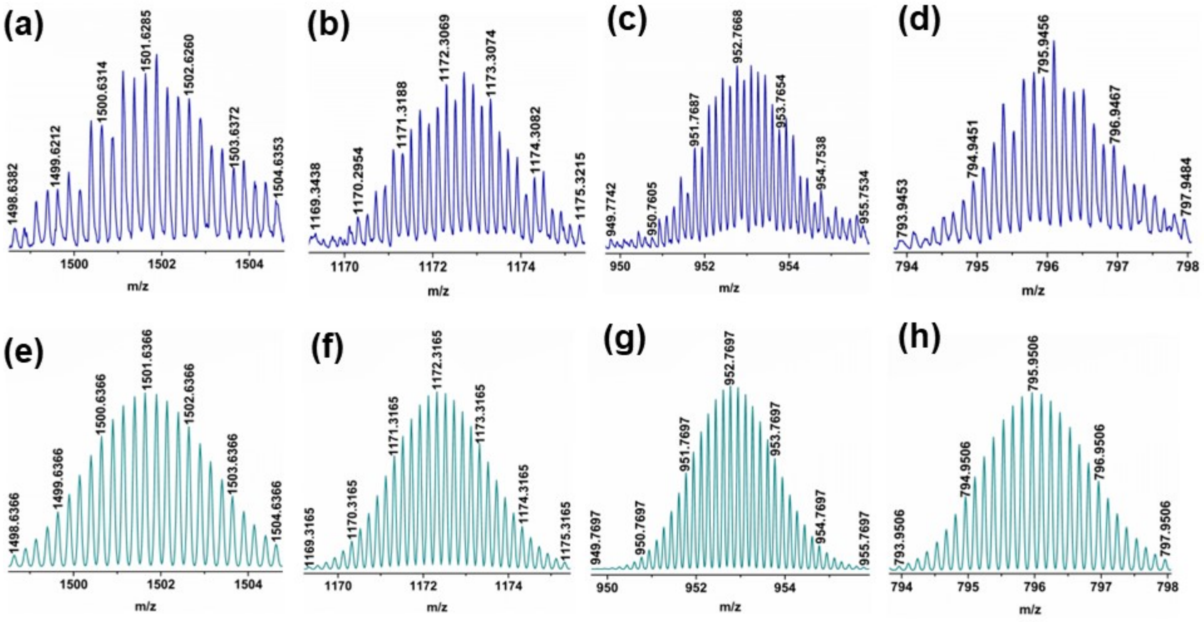

Fig. S17 Experimental (blue) isotopic distribution patterns of the mass-peaks corresponding to $[C_{60}⊂M_8L^{un}_4(PF_6)_{12}]^{4+}$ (a), $[C_{60}⊂M_8L^{un}_4(PF_6)_{11}]^{5+}$ (b), $[C_{60}⊂M_8L^{un}_4(PF_6)_{10}]^{6+}$ (c), and $[C_{60}⊂M_8L^{un}_4(PF_6)_9]^{7+}$ (d). Calculated (green) isotopic distribution patterns of the peaks corresponding to $[C_{60}⊂M_8L^{un}_4(PF_6)_{12}]^{4+}$ (e), $[C_{60}⊂M_8L^{un}_4(PF_6)_{11}]^{5+}$ (f), $[C_{60}⊂M_8L^{un}_4(PF_6)_{10}]^{6+}$ (g), and $[C_{60}⊂M_8L^{un}_4(PF_6)_9]^{7+}$ (h).

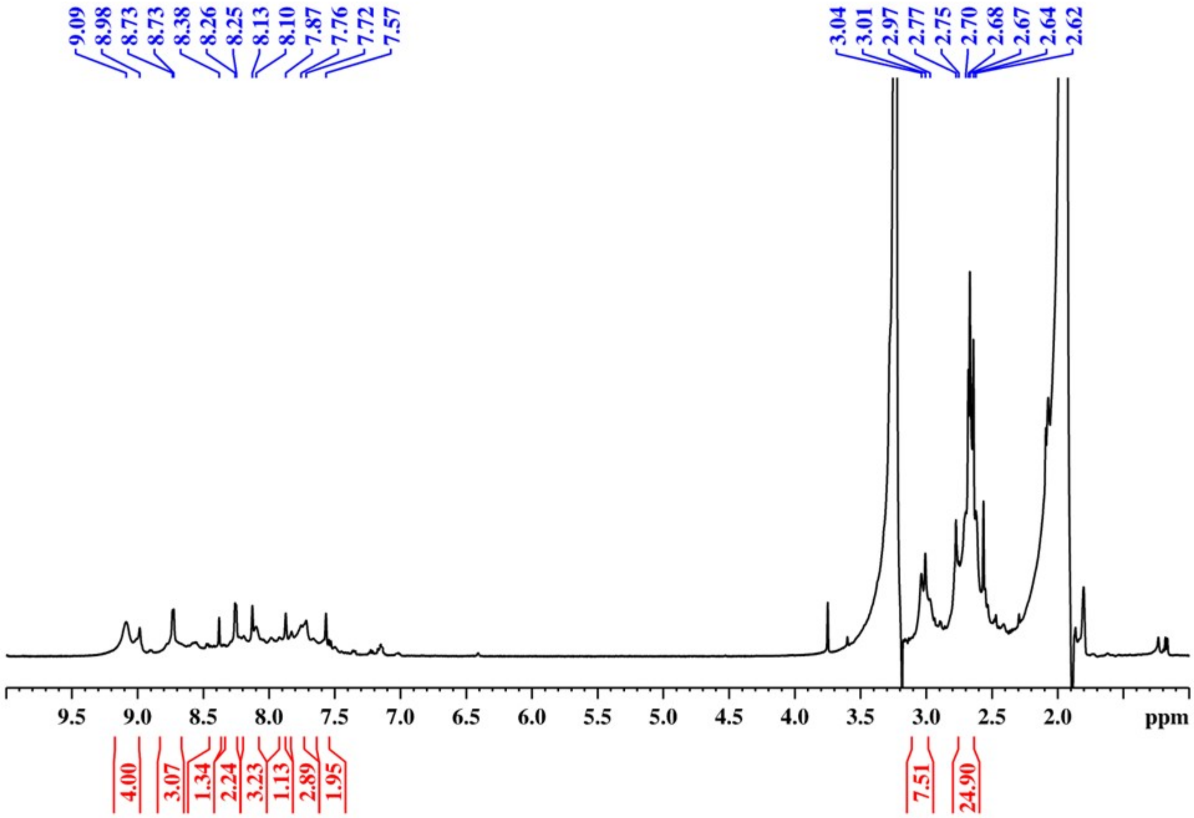

Fig. S18 ^{1}H NMR spectrum of $\mathbf{C_{70}{\subset}UNMB}$ recorded in CD_3CN (500 MHz, 298 K).

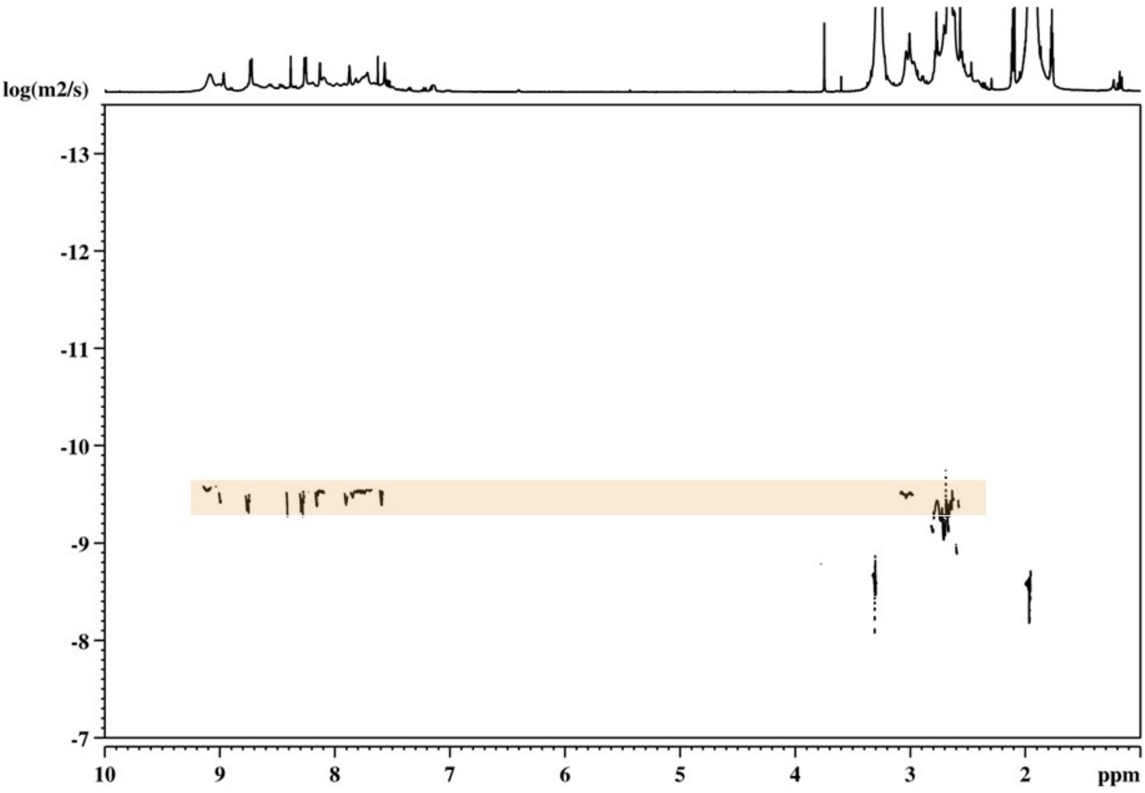

Fig. S19 2D DOSY NMR spectrum of $\mathbf{C_{70}{\subset}UNMB}$ recorded in CD_3CN (400 MHz, 298 K).

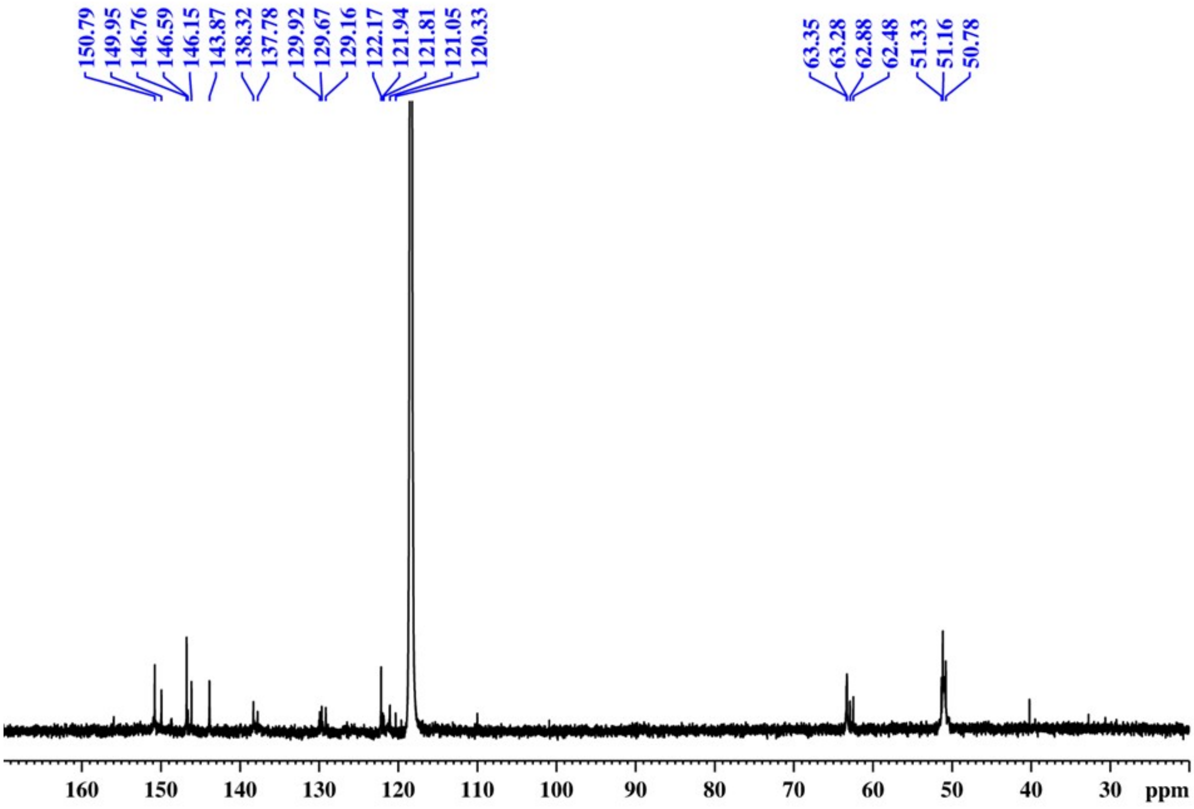

Fig. S20 ^{13}C NMR spectrum of $\mathbf{C_{70}{\subset}UNMB}$ recorded in CD_3CN (500 MHz, 298 K).

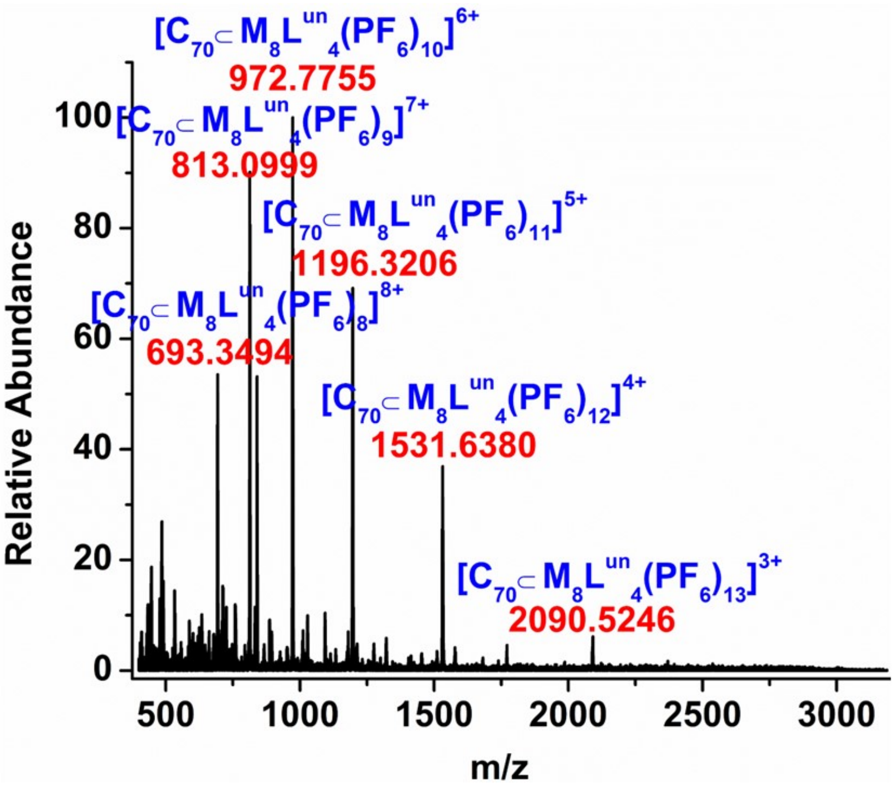

Fig. S21 ESI-MS spectrum of $\mathbf{C_{70}{\subset}UNMB}$ recorded in CH_3CN.

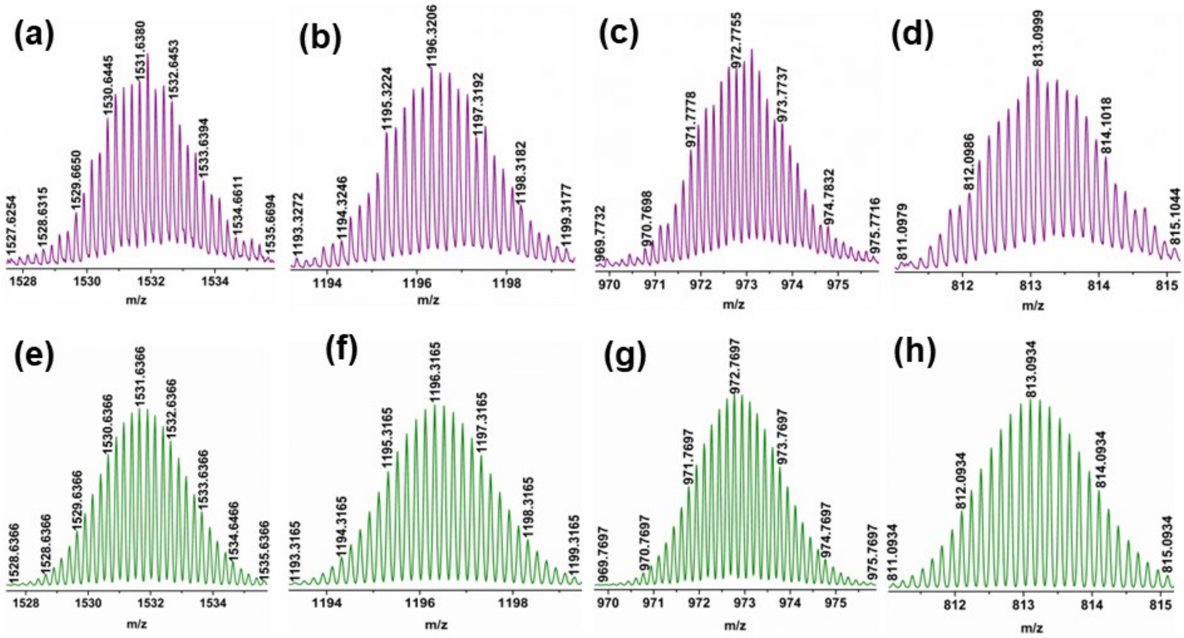

Fig. S22 Experimental (purple) isotopic distribution patterns of the mass-peaks corresponding to $[C_{70}\subset M_8L^{un}_4(PF_6)_{12}]^{4+}$ (a), $[C_{70}\subset M_8L^{un}_4(PF_6)_{11}]^{5+}$ (b), $[C_{70}\subset M_8L^{un}_4(PF_6)_{10}]^{6+}$ (c), and $[C_{70}\subset M_8L^{un}_4(PF_6)_9]^{7+}$ (d). Calculated (green) isotopic distribution patterns of the peaks corresponding to $[C_{70}\subset M_8L^{un}_4(PF_6)_{12}]^{4+}$ (e), $[C_{70}\subset M_8L^{un}_4(PF_6)_{11}]^{5+}$ (f), $[C_{70}\subset M_8L^{un}_4(PF_6)_{10}]^{6+}$ (g), and $[C_{70}\subset M_8L^{un}_4(PF_6)_9]^{7+}$ (h).

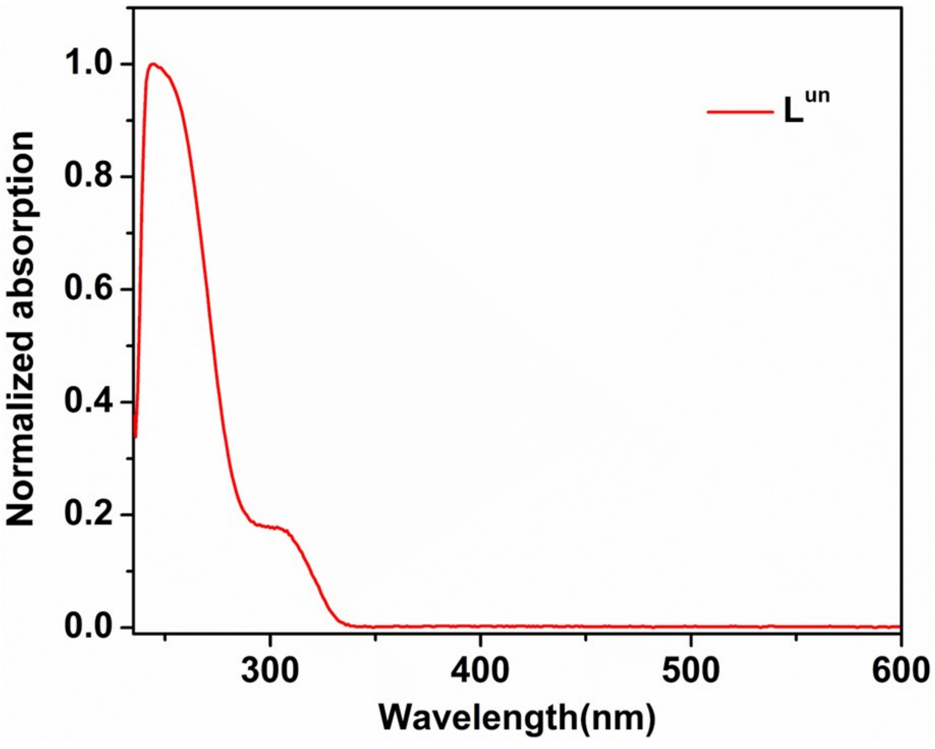

Fig. S23 UV-vis spectrum of $\mathbf{L^{un}}$ recorded in $CHCl_3$.

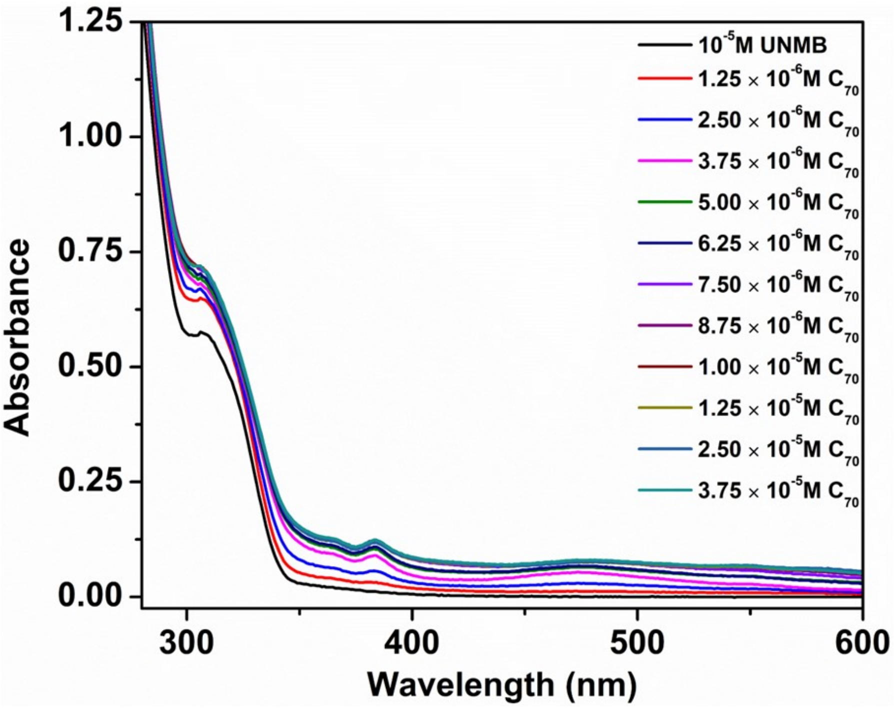

Fig. S24 UV-spectroscopic titration of acetonitrile solution of 1 (10 μM) with toluene solution of C70 (1 mM).

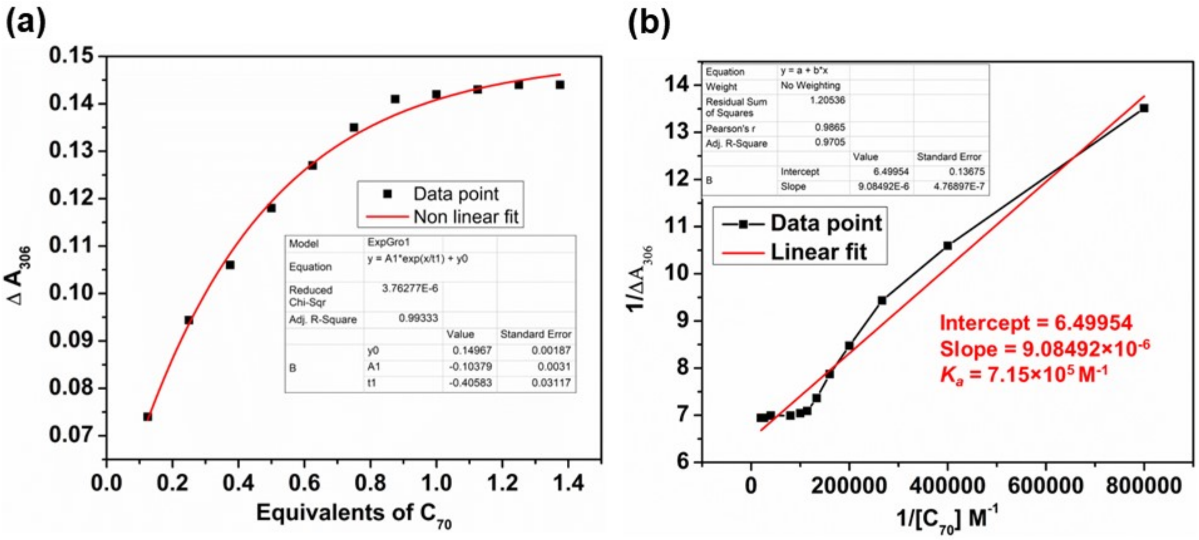

Fig. S25 Binding constant measurement of C_{70}⊂**UNMB**. (a) Plot of $\Delta A_{306\ nm}$ *vs* equiv. of C_{70} added; (b) Plot of $1/\Delta A_{306\ nm}$ *vs* $1/[C_{70}]$.

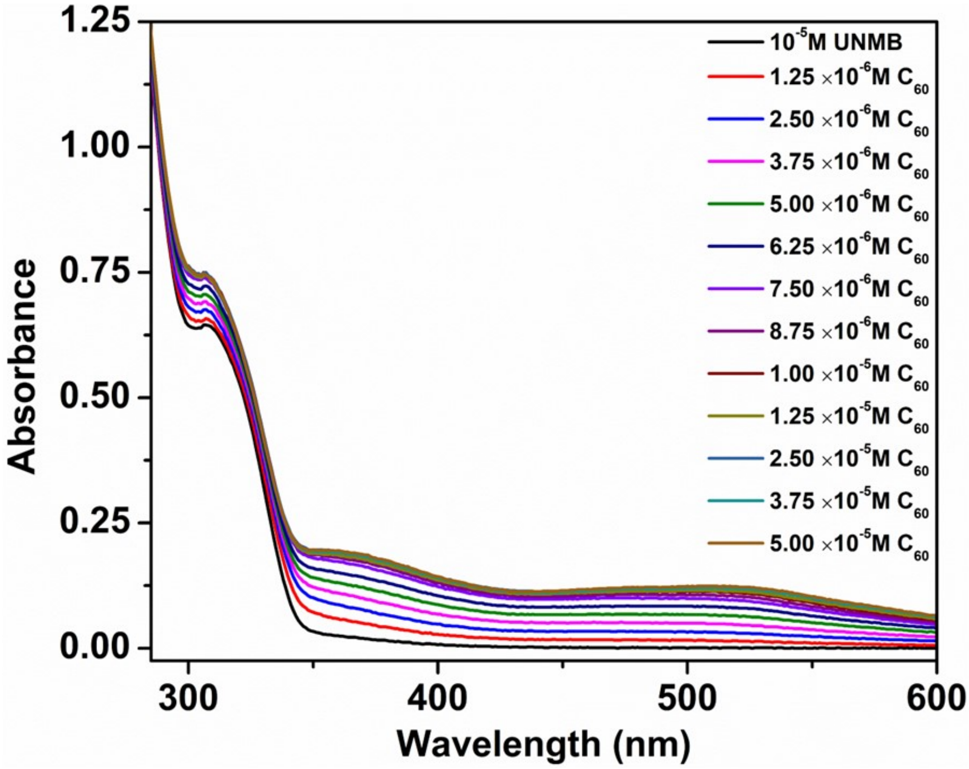

Fig. S26 UV-spectroscopic titration of acetonitrile solution of 1 (10μM) with toluene solution of C_{60} (1 mM).

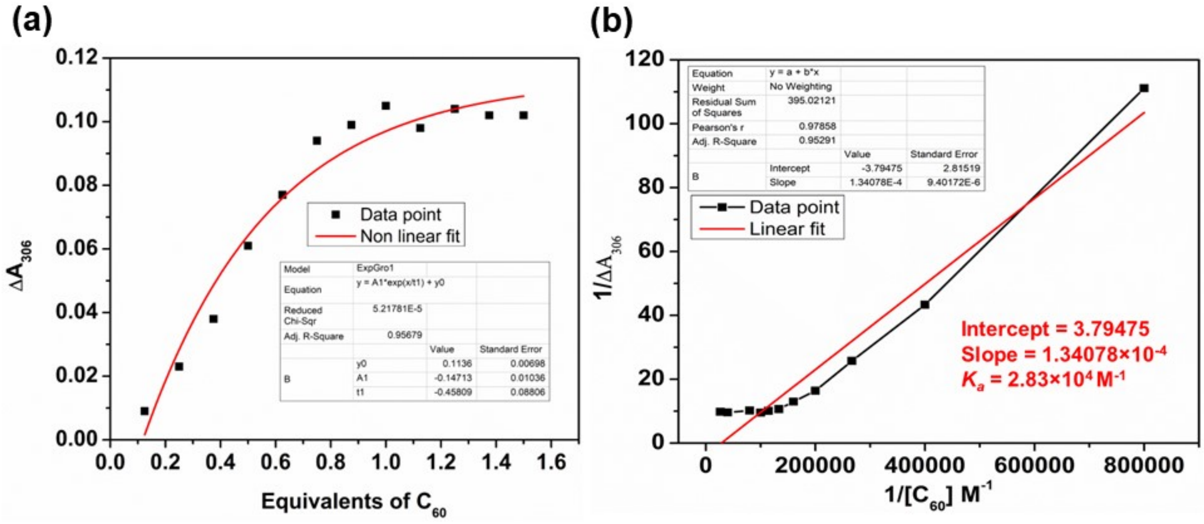

Fig. S27 Binding constant measurement of $C_{60}\subset$UNMB. (a) Plot of $\Delta A_{306\ nm}$ *vs* equiv. of C_{60} added; (b) Plot of $1/\Delta A_{306\ nm}$ *vs* $1/[C_{60}]$.

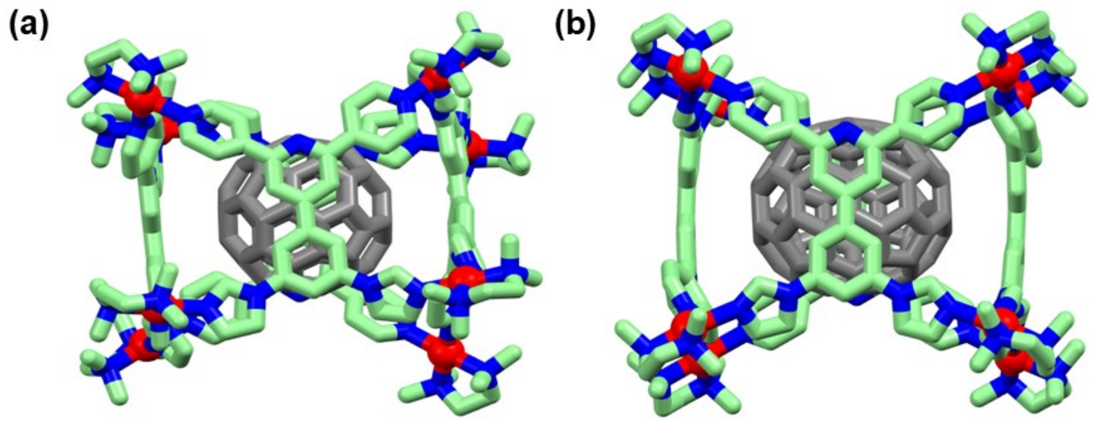

Fig. S28 DFT optimized structure of (a) **C_{60}⊂UNMB** (side view) and (b) **C_{70}⊂UNMB** (side view) showing π–π interaction between host and guest. Colour codes: carbon (light green for host and gray for guest), nitrogen (blue), palladium (red). H-atoms are omitted for clarity.

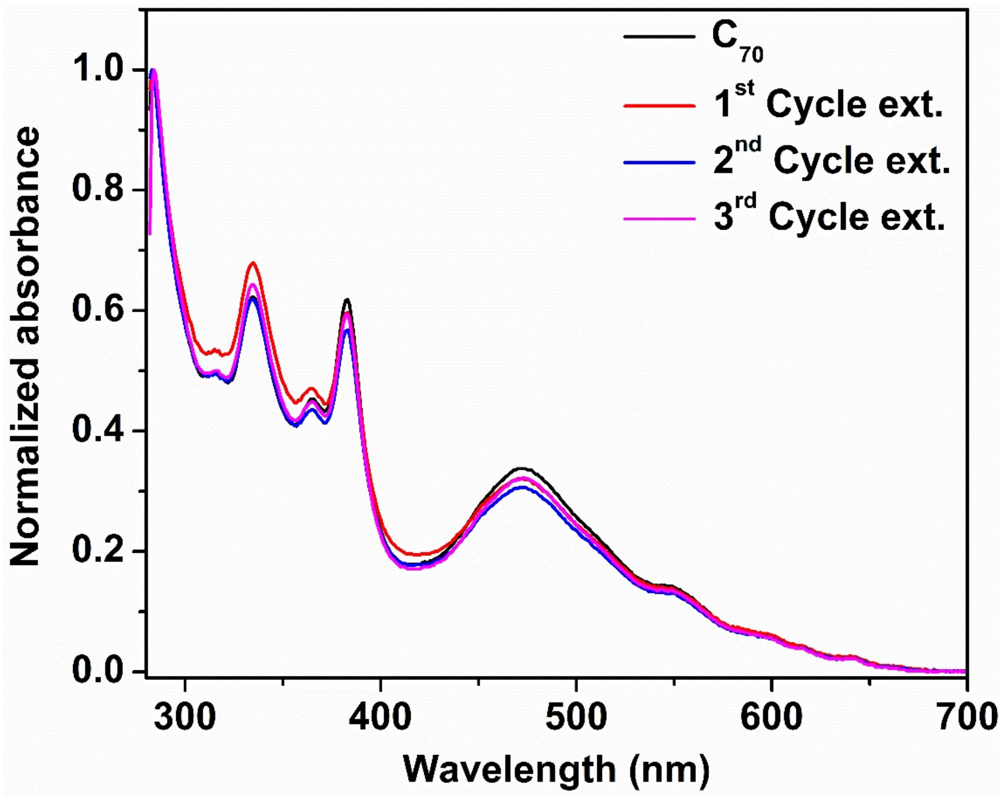

Fig. S29 Normalized absorption spectra of C_{70} and extracted guest after 1st, 2nd, and 3rd cycle (recorded in toluene 10^{-5} M).

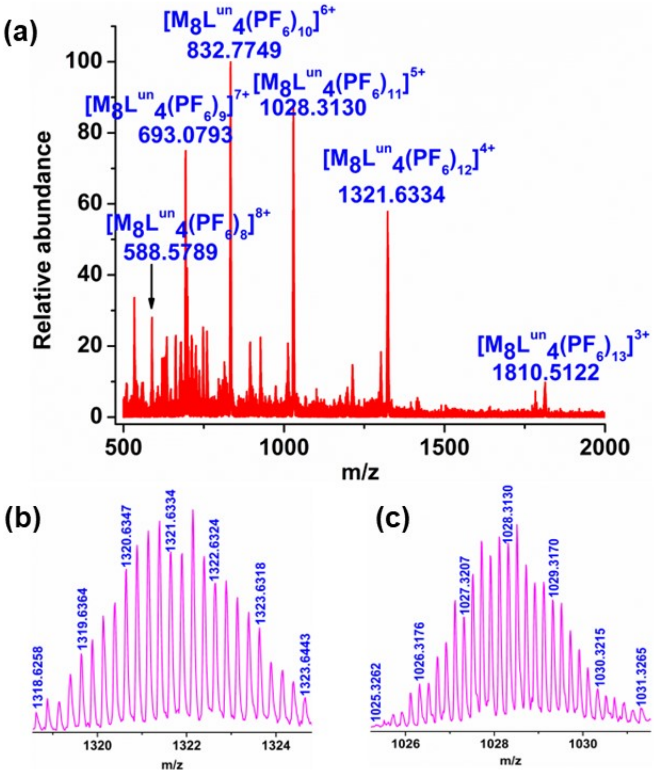

Fig. S30 ESI-MS of the recovered **UNMB**: Full ESI-MS spectrum in acetonitrile of the recovered **UNMB** after three cycles of C_{70} extraction (a); isotopic distribution patterns of the peaks correspond to fragments $[M_8L^{un}{}_4(PF_6)_{12}]^{4+}$ (b), and $[M_8L^{un}{}_4(PF_6)_{11}]^{5+}$ (c).

References

1. D. Prajapati, P. Bhandari, E. Zangrando and P. S. Mukherjee, A water-soluble Pd4 molecular tweezer for selective encapsulation of isomeric quinones and their recyclable extraction, *Chem. Sci.*, 2024, **15**, 3616-3624.
2. M. J. Frisch, G. W. Trucks, H. B. Schlegel, G. E. Scuseria, M. A. Robb, J. R. Cheeseman, G. Scalmani, V. Barone, B. Mennucci, G. A. Petersson, H. Nakatsuji, M. Caricato, X. Li, H. P. Hratchian, A. F. Izmaylov, J. Bloino, G. Zheng, J. L. Sonnenberg, M. Hada, M. Ehara, K. Toyota, R. Fukuda, J. Hasegawa, M. Ishida, T. Nakajima, Y. Honda, O. Kitao, H. Nakai, T. Vreven, J. A. Jr. Montgomery, J. E. Peralta, F. Ogliaro, M. Bearpark, J. J. Heyd, E. Brothers, K. N. Kudin, V. N. Staroverov, R. Kobayashi, J. Normand, K. Raghavachari, A. Rendell, J. C. Burant, S. S. Iyengar, J. Tomasi, MCossi, N. Rega, J. M. Millam, M. Klene, J. E. Knox, J. B. Cross, V. Bakken, C. Adamo, J. Jaramillo, R. Gomperts, R. E. Stratmann, O. Yazyev, A. J. Austin, R. Cammi, C. Pomelli, J. W. Ochterski, R. L. Martin, K. Morokuma, V. G. Zakrzewski, G. A. Voth,

P. Salvador, J. J. Dannenberg, S. Dapprich, A. D. Daniels, O. Farkas, J. B. Foresman, J. V. Ortiz, J. Cioslowski, D. J. Fox, Gaussian 16, Revision C. 01, *Gaussian. Inc., Wallingford CT* 2019.

3. R. OD, *CrysAlisPro*, Rigaku OD Yarton, Oxfordshire, UK, 2009-2021.
4. O. V. Dolomanov, L. J. Bourhis, R. J. Gildea, J. A. Howard, H. Puschmann, OLEX2: a complete structure solution, refinement, and analysis program *J. Appl. Cryst.* 2009, **42**, 339-341.
5. G. M. Sheldrick, SHELXT - Integrated space-group and crystal-structure determination. *Acta Cryst.* 2015**, A71**, 3-8.
6. G. M. Sheldrick, Crystal structure refinement with SHELXL. *Acta Cryst.* 2015, **C71 3-**8.
7. S. P. Vander, and A. L. Spek, BYPASS: an effective method for the refinement of crystal structures containing disordered solvent regions. *Acta Cryst.* 1990, **46**, 194.
